# SUMOylation of the ubiquitin ligase component KEAP1 at K39 upregulates NRF2 and its target function in lung cancer cell proliferation

**DOI:** 10.1016/j.jbc.2023.105215

**Published:** 2023-09-01

**Authors:** Hao Yang, Yuzhang Du, Xuefeng Fei, Shu Huang, Maimaitiaili Yimiti, Xiaobao Yang, Junrui Ma, Shuhui Li, Huxidanmu Tuoheniyazi, Yanan Zhao, Zhidong Gu, Dakang Xu

**Affiliations:** 1 Department of Laboratory Medicine, Ruijin Hospital, Shanghai Jiao Tong University School of Medicine, Shanghai, China; 2 College of Health Sciences and Technology, Shanghai Jiao Tong University School of Medicine, Shanghai, China; 3 Department of Laboratory Medicine, Ruijin-Hainan Hospital, Shanghai Jiao Tong University School of Medicine (Hainan Boao Research Hospital), Hainan, China

**Keywords:** SUMOylation, KEAP1, NRF2, cell proliferation, oxidative stress

## Abstract

Nuclear Factor Erythroid 2-Related Factor 2 (NRF2) is important for the expression of genes associated with oxidative stress. The levels of NRF2 are controlled by Kelch-like ECH-associated protein 1 (KEAP1)-dependent degradation. Although oxidative stress is known to suppress KEAP1 activity to stabilize the levels of NRF2, the mechanism for this control is unclear. Here, we identify that KEAP1 is modified by SUMO1 at the lysine residue position 39 (K39). Arginine replacement of this lysine (K39R) in KEAP1 did not affect its stability, subcellular localization, or dimerization but promoted the formation of the Cullin 3 ubiquitin ligase and increased NRF2 ubiquitination. This was accompanied by decreased NRF2 expression. Gene reporter assays showed that the transcription of antioxidant response elements was heightened in KEAP1-WT cells compared to cells expressing the KEAP1-K39R SUMO1 substrate mutant. Consistent with this, chromatin immunoprecipitation assays revealed higher NRF2 binding to the promoter regions of antioxidant genes in cells expressing the KEAP1-WT compared to the KEAP1-K39R mutant protein in H1299 lung cancer cell. The significance of this suppression of KEAP1 activity by its SUMOylation was tested in a subcutaneous tumor model of H1299 lung cancer cell lines that differentially expressed the WT and K39R KEAP1 constructs. This model showed that mutating the SUMOylation site on KEAP1 altered the production of reactive oxygen species and suppressed tumor growth. Taken together, our study recognizes that NRF2-dependent redox control is regulated by the SUMOylation of KEAP1. These findings identify a potential new therapeutic option to counteract oxidative stress.

As a transcription factor, the Nuclear factor erythroid 2-related factor 2 (NRF2) regulates oxidative stress and is crucial to maintaining redox homeostasis (1). Under normoxic conditions, NRF2 expression is suppressed by Kelch-like ECH-associated protein 1 (KEAP1). Under oxidative stress, NRF2 is released from KEAP1 and translocates to the nucleus where it binds to the antioxidant response element (ARE) sequence in gene promoters (2, 3). This regulation of NRF2 is thought to account for the observed pathogenesis of KEAP1 mutations. Point mutations in the central intervening region (IVR) or in the Kelch domain of KEAP1 that alter its interaction with NRF2 have been reported in non-small cell lung cancer cells (NSCLC) (4, 5). Inactivating mutations of KEAP1 led to NRF2 accumulation and hyperactivation of NRF2 target genes that promoted tumor cell growth (5, 6, 7). Accordingly, there is motivation to better understand the processes that regulate the NRF2-dependent response to identify therapeutic options to treat hypoxic conditions.

KEAP1 forms part of an E3 ubiquitin ligase, which tightly regulates the activity of NRF2 by targeting it for ubiquitination and proteasome-dependent degradation. KEAP1 promotes proteasome-mediated degradation of NRF2 through a Cullin 3 (CUL3) based E3 ubiquitin ligase complex (8). Here, we identify that KEAP1 activity is controlled by its modification with one of the Small ubiquitin-like modifier (SUMO) proteins.

The reversible modification of proteins by SUMOs plays a key role in various cellular processes (9). In the SUMOylation-mediated cascade, SUMO is activated by an E1 activating enzyme and covalently transferred to an E2 ligase UBC9, which is the only E2 ligase for all SUMOs (SUMO1, 2 and 3 are functionally important). UBC9 interacts with various substrates to transfer SUMO to lysine residues in target proteins aided by E3 ligases (10). Interestingly, it has been reported that modification of NRF2 by SUMOs induces its release from KEAP1 and affects its nucleocytoplasmic localization, stability, and transcriptional activity (2, 11, 12, 13).

In this study, we identified that KEAP1 was modified by SUMO1. SUMOylation of KEAP1 suppressed its control of NRF2. This did not affect the association between KEAP1 and NRF2 but appeared to alter the CUL3 ubiquitination complex and decrease NRF2 ubiquitination. This increased the protein levels with expression of NRF2-regulated genes. We determined a SUMOylation site on KEAP1 and demonstrated that mutating this residue ablated its control of the growth of human NSCLC cells *in vitro* and *in vivo*. These results identify that SUMOylation of KEAP1 regulates the physiological functions of NRF2.

## Results

### KEAP1 is modified by SUMO1

**Figure 1:**
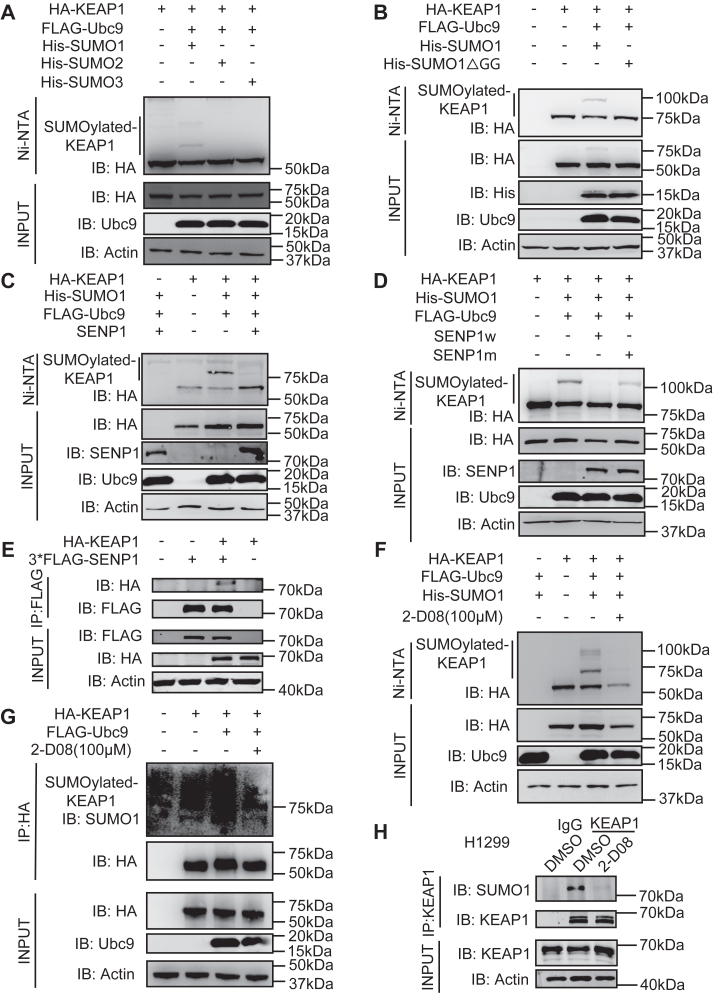
**KEAP1 is modified by SUMO1 and deSUMOylated by SENP1.***A* and *B*, WB analysis of SUMOylated KEAP1 in the lysates of HEK-293T cells transfected with plasmids expressing HA-tagged KEAP1 and His-tagged SUMO1/2/3 (*A*) or mutant SUMO1 (ΔGG) (*B*) with anti-HA antibody after IP with Ni_2_^+^-NTA. *C* and *D*, WB analysis of SUMOylated KEAP1 in the lysates of HEK-293T cells overexpressing FLAG-SENP1 (*C*) or FLAG-SENP1w (WT), FLAG-SENP1m (Mutant), (*D*) and HA-KEAP1, His-SUMO1, and FLAG-UBC9 with anti-HA antibody after IP with Ni_2_^+^-NTA. *E*, WB analysis of KEAP1 and SENP1 in the lysates of HEK-293T cells overexpressing HA-KEAP1-WT and FLAG-SENP1 after IP with anti-FLAG. *F*, WB analysis of SUMOylated KEAP1 in the lysates of HEK-293T cells overexpressing HA-KEAP1 or UBC9 and SUMO1 with the DMSO solvent (−) or the inhibitor 2-D08 after IP with Ni_2_^+^-NTA. *G*, WB analysis of SUMOylated KEAP1 in the lysates of HEK-293T cells overexpressing HA-KEAP1 and UBC9 with or without DMSO or 2-D08 after IP with anti-HA beads. Representative results from three independent experiments are shown. *H*, A confirmation of the SUMOylation of endogenous KEAP1 in H1299 cells by IP with anti-KEAP1 or anti-IgG antibodies then IB with an anti-SUMO1 antibody.

To determine whether KEAP1 is a SUMO substrate, we transiently transfected HEK-293T cells with plasmids expressing HA-tagged KEAP1, FLAG-tagged UBC9, and His-tagged SUMO1, SUMO2, or SUMO3. Precipitation of His-SUMO conjugates by Ni_2_^+^-NTA resin followed by immunoblotting assays was performed as described in our previous experiments (14). These experiments identify that KEAP1 was modified by SUMO1 but not by SUMO2 or SUMO3 (Fig. 1*A*). The specificity of this assay was verified by repeating the experiment using a mutant SUMO1 that had the diglycine residues that form the thioester linkage deleted. This abolished the apparent SUMOylation of KEAP1 in this assay (Fig. 1*B*). SUMOylation is a reversible process and SUMO1 can be removed by the Sentrin/SUMO-specific protease 1 (SENP1) (15). Therefore, we validated KEAP1 modification by SUMO1 by demonstrating its deSUMOylated by transiently transfected 293T cells with plasmids expressing HA-KEAP1, His-SUMO1, and FLAG-SENP1 or as a control inactive mutant SENP1 construct (Fig. 1, *C* and *D*). Immunoprecipitation with immunoblot appears to confirm the interaction between SENP1 and KEAP1 (Fig. 1*E*). Moreover, the SUMOylation of KEAP1 was suppressed by treatment with a pharmacological inhibitor (2-D08 (16)) (Fig. 1, *F* and *G*). SUMOylation of the endogenous levels of KEAP1 was demonstrated by immunoblotting peptides, which were enriched with an anti-KEAP1 antibody from human H1299 lung carcinoma cells untreated or treated with 2-D08, with an anti-SUMO1 antibody (Fig. 1*H*). Taken together, these results identify that KEAP1 was covalently modified with SUMO1.

### The K39R on KEAP1 is SUMOylated

**Figure 2:**
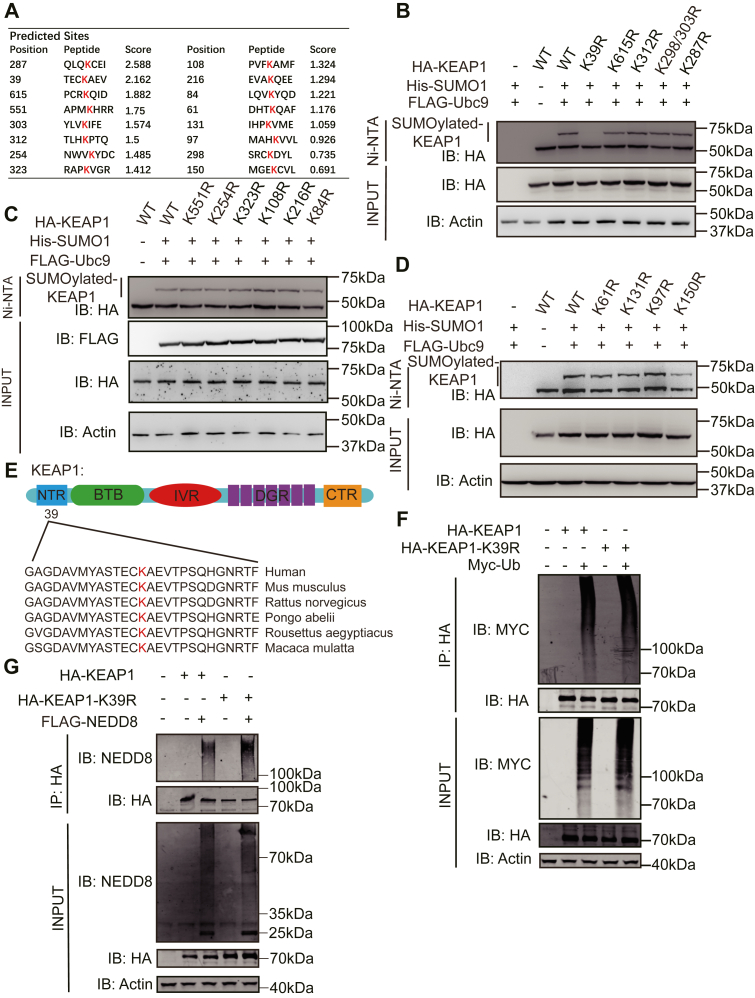
**The lysine residue at position 39 on KEAP1 is SUMOylated**. *A*, the 16 candidate SUMOylation sites in KEAP1 predicted by the SUMOsp 2.0 software. *B–D*, WB analysis of SUMOylated KEAP1 IP with an anti-HA antibody from the lysates of HEK-293T cells overexpressing His-SUMO1 with HA-KEAP1 WT and arginine replacement mutants of predicted SUMOylated residues. *E*, sequence alignment of KEAP1 peptides from the indicated species. The predicted SUMOylated K39 site is indicated as a red letter. *F*, measures of the ubiquitination of the lysine residues at position K39 on KEAP1 in HEK-293T cells expressing Myc-tagged ubiquitin (Myc-Ub) with either HA-tagged WT or mutant (K39R) KEAP1, followed by IP with an anti-HA antibody and IB with anti-Myc antibody. *G*, measures of KEAP1 neddylation by IB of proteins IP from HEK-293T cells transfected with indicated constructs with an anti-HA antibody with an anti-NEDD8 antibody. Representative results from three independent experiments are shown.

A prediction tool was used to identify putative SUMOylation sites on KEAP1 (SUMOsp 2.0 (17, 18)) (Fig. 2*A*). Arginine replacement mutagenesis was conducted to test which of these sites were modified by SUMO1. The primers used for the construction of KEAP1 mutants are listed in Table S1. HEK-293T cells were transfected with plasmids expressing SUMO1 and UBC9 with either the WT or the mutant KEAP1 constructs and then the levels of SUMOylation were measured as performed previously. This identified the K39 of KEAP1 as a bona fide SUMOylation site (Fig. 2, *B*–*D*). Notably, this site appears to be highly conservation in mammals (Fig. 2*E*).

Because it has been speculated that SUMOylation counteracts ubiquitin-mediated effects, we sought to assess competition for this K39 lysine residues by SUMO1 and ubiquitin (Ub). Towards this, the WT or mutant KEAP1 (K39R) was expressed in HEK-293T cells Ub. Immunoblotting of immunoprecipitated WT and KEAP1 (K39R) with an anti-Ub antibody detected an equivalent signal, suggesting these separate posttranslational modifications do not compete for this residue (Fig. 2*F*). We also tested if the K39 lysine on KEAP1 was modified with the ubiquitin-like Neural precursor cell expressed developmentally down-regulated 8 (NEDD8), as this modification controls other components of the NRF2 ubiquitination complex. No difference in NEDDylated products was apparent in peptides enriched with KEAP1 from cells expressing the WT or K39R protein (Fig. 2*G*). Together, our results indicated that the lysine residue at position 39 on KEAP1 is modified by SUMO1.

### Mutation of the lysine residue 39 on KEAP1 does not alter protein stability, localization, or dimerization

**Figure 3:**
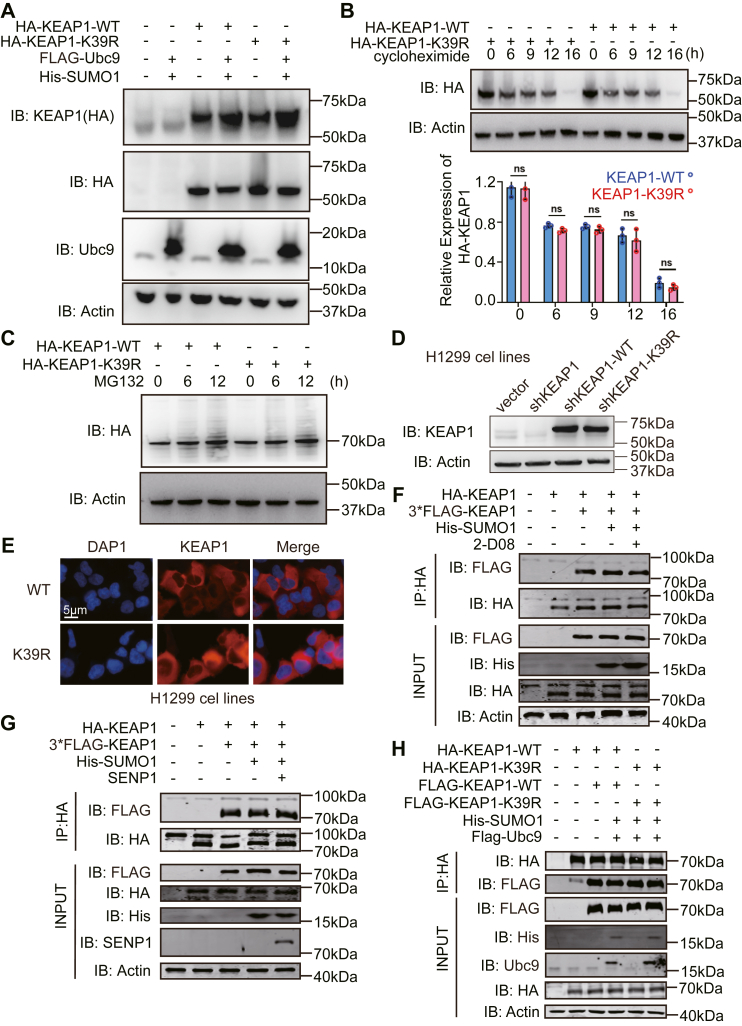
**The K39 residue does not affect KEAP1 stability or cellular location**. *A*, WB analysis of KEAP1 in the lysates of HEK-293T cells overexpressing HA-KEAP1-WT or HA-KEAP1-K39R with or without His-SUMO1 and FLAG-UBC9. *B*, WB analysis of KEAP1 in the lysates of HEK-293T cells overexpressing HA-KEAP1-WT or HA-KEAP1-K39R with or without 0.1 mg/ml cycloheximide treatment during the indicated time. *C*, WB analysis of KEAP1 in the lysates of HEK-293T cells overexpressing HA-KEAP1-WT or HA-KEAP1-K39R treated with the DMSO solvent or 20 μM MG132 at different time points. *D*, WB analysis to detect the KEAP1 protein expression at indicated molecules in H1299 cells stably transfected with shKEAP1, KEAP1-WT, or KEAP1-K39R. *E*, confocal microscopy analysis of KEAP1 in H1299-shKEAP1-WT or H1299-shKEAP1-K39R cells. *F*, measures of the dimerization of KEAP1 in HEK-293T cells expressing FLAG and HA-tagged KEAP1 and SUMO1 or treated with the SUMOylation Inhibitor 2-D08 by IP with an anti-FLAG (*upper panel*) or anti-HA (*lower panel*) antibodies then IB with the reciprocal antibody. *G*, detection of the effect of SUMOylation for KEAP1 dimerization in HEK-293T cells transfected with SUMO1, SENP1 and KEAP1 tagged with FLAG or HA, then IP with an anti-HA antibody followed by IB of SDS-PAGE gel separated immune-enriched proteins with an anti-FLAG antibody. *H*, measures of the effect of arginine replacement of the lysine residues at positions K39 for KEAP1 dimerization, as previously performed. Representative results from three independent experiments are shown.

Experiments were conducted to assess the function of the lysine residue at position 39 on KEAP1. Although the preceding data didn’t detect an effect on the ubiquitination of KEAP1, we proceeded to test if the K39R mutation altered KEAP1 degradation. HEK293T cells were transfected with KEAP-WT or KEAP1-K39R with or without SUMO1 and UBC9 and the relative levels of the proteins were compared by immunoblot. Consistent with the data shown in Figure 2*F*, there was no measurable effect of preventing SUMOylation at K39 for the levels of KEAP1 (Fig. 3*A*). The protein half-life of the WT and K39R mutant KEAP1 was also not altered in cells following cycloheximide treatment to inhibit protein synthesis (Fig. 3*B*). Moreover, the K39R mutation did not significantly alter the protein level of KEAP1 with MG132 treatment to inhibit proteasome-dependent degradation (Fig. 3*C*). To alternatively assess if the K39 residue altered the expression of KEAP1, we constructed human H1299 lung carcinoma cells with knockdown of the endogenous KEAP1 by expression of a short-hairpin RNA, then introduced constructs to re-express either the WT or KEAP1-K39R proteins (coded H1299-shKEAP1-WT or -K39R, respectively). Western blotting (WB) showed no obvious differences in protein expression of the WT and KEAP1-K39R proteins (Fig. 3*D*). These findings appear to demonstrate that SUMOylation does not alter the expression or stability of KEAP1.

SUMOylation is also known to regulate substrate intracellular distribution (19), so we investigated whether KEAP1 cellular localization was affected by SUMOylation by mutating the K39 residue on KEAP1. Towards this, H1299 cells were transfected with KEAP1-WT or the K39R mutant then the proteins were visualized by immunofluorescent staining with an anti-KEAP1 antibody. This showed that both proteins were mainly located in the cytoplasm and detected no apparent differences (Fig. 3*E*). Accordingly, SUMOylation does not appear to alter the cellular localization of KEAP1.

Dimerization of KEAP1 is required for its association with NRF2 (20). To test if SUMOlyation impacted the formation of KEAP1 homodimers we transfected HEK-293T cells with differently tagged KEAP1 constructs and then assessed if the indirect capture of a FLAG-KEAP1 by immune-enrichment of HA-KEAP1 was altered by SUMOylation. This was tested by co-expressing SUMO1 with and without treatment with a SUMOylation Inhibitor (2-D08). Immunoblotting of immunoprecipitated peptides demonstrates no significant differences in the recovery of the differently tagged WT and K39R KEAP1 proteins (Fig. 3*F*). As an alternative approach, we repeated this experiment but co-expresed SENP-1 to deSUMOylate KEAP1. This also didn’t affect the indirect recovery of the HA-KEAP1 and FLAG-KEAP1 proteins (Fig. 3*G*). A function for the lysine residues at position 39 was tested in the same way by comparing the recovery of differently tagged WT and K39R KEAP1 proteins (Fig. 3*H*). These data indicated that the SUMOylation doesn’t alter the dimerization of the KEAP1.

### SUMOylation of KEAP1 inhibits the ubiquitination-dependent degradation of NRF2

**Figure 4:**
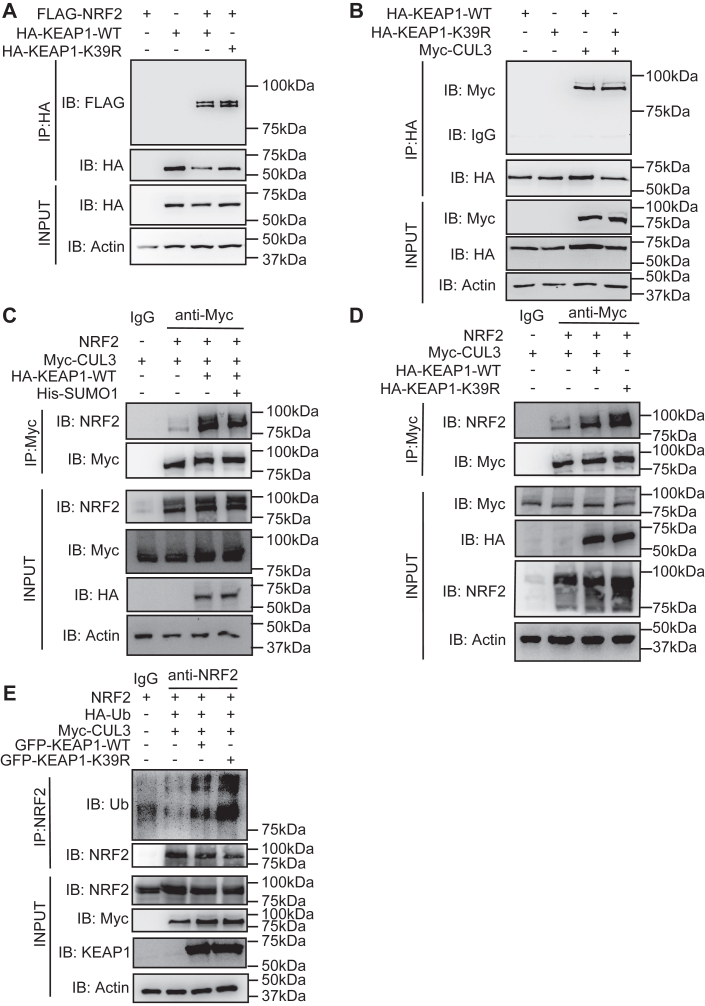
**SUMOylation of KEAP1 inhibits NRF2 ubiquitination-dependent degradation**. *A* and *B*, WB analysis of the interaction between KEAP1 and NRF2 (*A*) or KEAP1 and CUL3 (*B*) in the lysates of HEK-293T cells overexpressing HA-KEAP1-WT or HA-KEAP1-K39R and FLAG-NRF2 or Myc-CUL3 and treated with 20 μM MG132 after IP with an anti-HA and IB with anti-FLAG or anti-Myc antibodies. *C* and *D*, WB analysis of Myc-CUL3 and NRF2 in the lysates of HEK-293T cells overexpressing HA-KEAP1 and His-SUMO1 (*C*), or HA-KEAP1-WT or HA-KEAP1-K39R (*D*) after IP with anti-Myc and IB with anti-NRF2 antibodies. *E*, WB analysis of NRF2 ubiquitination in the lysates of HEK-293T cells overexpressing GFP-KEAP1-WT or GFP-KEAP1-K39R and NRF2, HA-Ub, and Myc-CUL3 treated with 20 μM MG132 after IP with an anti-NRF2 antibody. Representative results from three independent experiments are shown.

KEAP1 directly binds to NRF2 to hinder its nuclear entry and affect its ubiquitination and degradation (21). Accordingly, we investigated how KEAP1 SUMOylation affected the formation of the NRF2 ubiquitination complex. HEK-293T cells were transfected with NRF2 and the WT or KEAP1-K39R constructs, then KEAP1 was IP and the enriched proteins were probed for the indirect recovery of NRF2. This shows that mutation of the K39 residue did not affect the interaction between KEAP1 and NRF2 (Fig. 4*A*). Replication of this experiment by expressing KEAP1 with CUL3 also demonstrated that the K39 residue didn’t affect KEAP1’s association with the ubiquitin ligase (Fig. 4*B*). Expression of the three factors along with SUMO1, followed by IP shows that KEAP1 promoted the interaction of NRF2 with CUL3 and that this was weakened with SUMO1 overexpression (Fig. 4*C*). Correspondingly, the inhibitory effect of SUMO1 on the interaction of CUL3 and NRF2 was lost by mutating the K39 residue on KEAP1 (Fig. 4*D*). Furthermore, the KEAP1-K39R increased the ubiquitination of NRF2 compared with KEAP1-WT (Fig. 4*E*). These data suggest that the SUMOylation of KEAP1 inhibits NRF2 ubiquitination by disrupting the assembly of an effective ubiquitin complex. Additional experiments are required to identify precisely how the covalently linked SUMO1 causes this disruption.

### KEAP1 SUMOylation promotes NRF2-target gene expression and reduces oxidative stress

**Figure 5:**
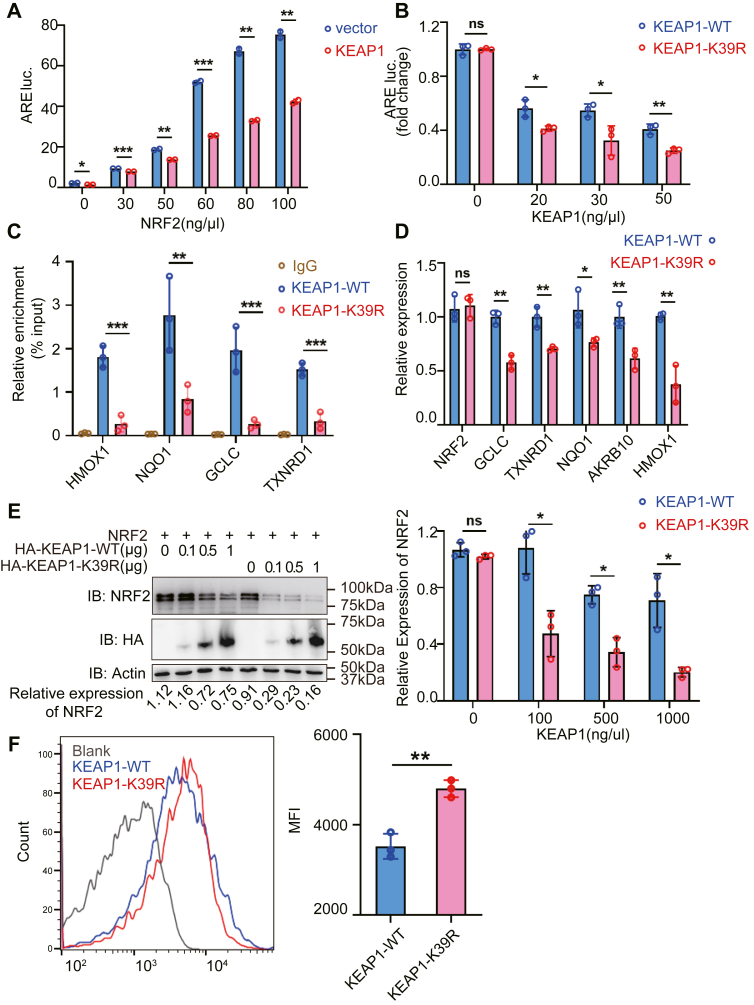
**SUMOylation of KEAP1 promotes NRF2-targeted gene expression and reduces ROS**. *A*, luciferase activity in HEK-293T cells transfected with ARE-luciferase and constitutive Renilla reporters, NRF2 and KEAP1 expression constructs or the empty vector for 12 h, normalized to Renilla luciferase activity (*n* = 2). *B*, luciferase assay of HEK-293T cells transfected with the ARE-luciferase and constitutive Renilla reporters and expressing NRF2 and KEAP1-WT or KEAP1-K39R for 12 h. Results are expressed as the fold ratio above the cells transfected with KEAP1-WT or KEAP1-K39R, normalized to Renilla luciferase activity (*n* = 3). *C*, measures of the indicated gene sequences that were enriched by IP with an anti-NRF2 antibody from H1299 cells with knockdown of KEAP1 and re-expressing the WT or K39R mutant KEAP1. *D*, measures of the levels of the indicated antioxidant genes in H1299-shKEAP1-WT and H1299-shKEAP1-K39R cells were measured by qRT-PCR (*n* = 3). *E*, WB analysis of the levels of NRF2 in the lysates of HEK-293T cells transfected with NRF2 and the indicated amounts of HA-KEAP1-WT or HA-KEAP1-K39R with anti-HA and anti-NRF2 antibodies. The levels of the NRF2 signals relative to Actin is shown below the blot. *F*, measures of the level of ROS in H1299-shKEAP1-WT and H1299-shKEAP1-K39R cells. A representative result from three independent experiments is shown (n = 3). The graphs show the mean ± s.d. *p* values are calculated by Student’s *t* test (ns = not significant; ∗*p* < 0.05; ∗∗*p* < 0.01; and ∗∗∗*p* < 0.001).

Given that SUMOylated KEAP1 inhibited NRF2 ubiquitination-dependent degradation, we wondered whether SUMOylated KEAP1 promoted NRF2-targeted gene expression to control the production of cellular reactive oxygen species (ROS) (22). We first confirmed that NRF2 induced a luciferase reporter gene controlled by the antioxidant response element (ARE). Towards this, HEK-293T cells were transfected with ARE firefly and constitutive Renilla luciferase reporters and plasmids expressing KEAP1 with increasing amounts of NRF2. This confirms that NRF2 controls the induction of the ARE promoter element in a dose-dependent manner under constraint by KEAP1 (Fig. 5*A*). In this assay, inhibiting the SUMOylation of KEAP1 by mutating the K39 residue further reduced NRF2-dependent induction of the ARE promoter element (Fig. 5*B*). Next, we assessed NRF2-binding to endogenous gene promoters by chromatin immunoprecipitation (ChIP) assay. Chromatin enriched with an anti-NRF2 antibody from cells expression of the WT or KEAP1-K39R proteins were amplified with oligonucleotides for the NRF2 regulated *NQO1*, *HMOX1*, *GCLC*, and TXNRD1 gene promoters. The ChIP signals were compared against that generated by an anti-IgG antibody (considered as background) and presented as the fold increase in signal relative to the background signal. The results show that NRF2 binding to gene promoters was significantly higher in the cells expressing the WT compared with the KEAP1-K39R protein (Fig. 5*C*). Furthermore, we measured the effect of mutating the K39 residue on KEAP1 for the levels of NRF2-regulated antioxidant transcripts (23). The data showed that the levels of the *NQO1*, *HMOX1*, *GCLC*, *TXNRD1*, and *AKR1B10* transcripts were lower in cells expressing KEAP1-K39R compared with KEAP1-WT (Fig. 5*D*). As expected, overexpressing KEAP1-K39R induced a greater reduction in the levels of NRF2 compared to KEAP1-WT as visualized by immunoblot (Fig. 5*E*). In keeping with the reduction in NRF2 levels and ensuing decreased expression of dependent genes, KEAP1-K39R overexpression markedly increased the production of ROS (Fig. 5*F*). These data demonstrate that the SUMOylation of KEAP1 suppressed its constraint of the levels of NRF2, thereby promoting the expression of NRF2-targeted genes to limit ROS production.

### Inhibiting the SUMOylation of KEAP1 suppressed tumor growth

**Figure 6:**
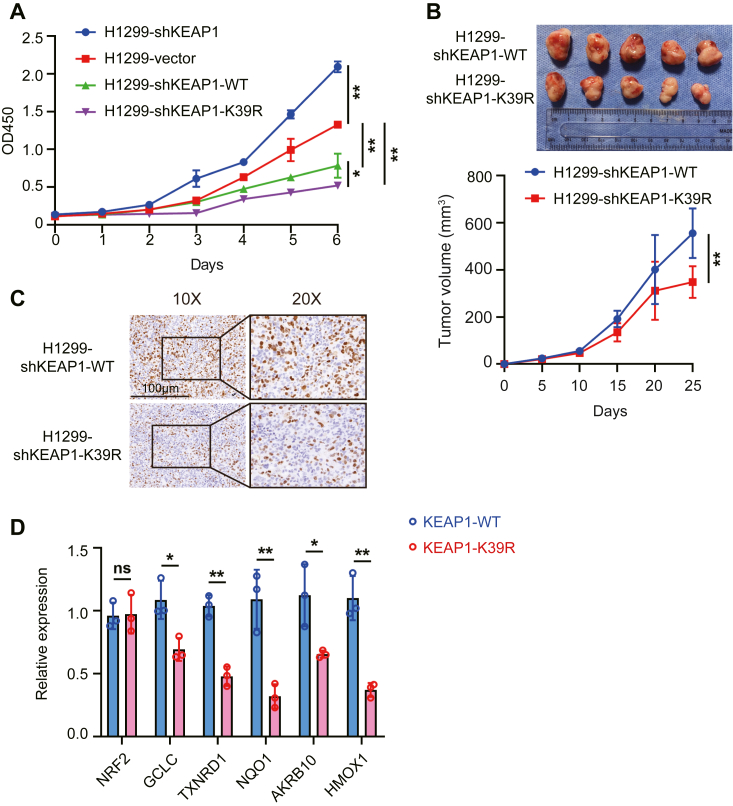
**KEAP1 SUMOylation promotes tumor growth**. *A*, the effect of KEAP1 on cell proliferation *in vitro* is assessed by comparing the H1299-shKEAP1, H1299-shKEAP1-WT and H1299-shKEAP1-K39R or as a control H1299 cell transfected with the empty vector cell lines by a CCK8 cell proliferation assay. *B* and *C*, the effect of re-expressing KEAP1-WT or KEAP1-K39R in the H1299-shKEAP1 cell line for tumor growth was assessed *in vivo* by injecting 2.5 × 10^6^ of each cell subcutaneously into male BALB/c nude mice then 35 days later dissecting the tumors (shown at the top) and their volume measured (*B*). *C*, detection of Ki67 by immunohistochemistry staining in xenograft tumors. *D*, measures of the levels of the indicated antioxidant transcripts in xenograft tumors measured by qRT-PCR (*n* = 3). The graphs show the mean ± s.d. *p* values are calculated by Student’s *t* test (ns = not significant; ∗*p* < 0.05; ∗∗*p* < 0.01; and ∗∗∗*p* < 0.001).

As the KEAP1-NRF2 pathway was found to be highly related to tumor progression (24), we assessed the impact of KEAP1 SUMOylation on the proliferation of the NSCLC H1299 cell line. Toward this, the expression of endogenous KEAP1 was ablated by RNA interference and then the WT or K39R mutant KEAP1 was re-expressed in cells (coded H1299-shKEAP1, H1299-shKEAP1-WT, and H1299-shKEAP1-K39R, respectively). A comparison of the growth rate of these cells showed that KAEP1 slows the rate of proliferation and identified that disruption of KEAP1 SUMOylation at the K39 residue reinforces this (Fig. 6*A*) (25). We next assessed these cell lines in a xenografted murine model to explore the effect of KEAP1 SUMOylation for tumor growth *in vivo.* Compared with that in H1299-shKEAP1-WT tumor-bearing mice the growth of the H1299-shKEAP1-K39R tumors was greatly retarded (Fig. 6*B*). Immunohistochemical staining of tumors from mice with the Ki67 proliferation marker confirmed reduced proliferation of cells in tumors expressing the K39R mutant compared to the WT KEAP1 (Fig. 6*C*). Measures of the levels of the anti-oxidative *NQO1*, *HMOX1*, *GCLC*, *TXNRD1*, and *AKR1B10* transcripts were also lower in tumors expressing the K39R mutant compared to the WT KEAP1 (Fig. 6*D*). These data support the effect of SUMOylation as repressing KEAP1’s regulation of NRF2 *in vivo*.

Overall, these findings identify a previously unrecognized SUMOylation of KEAP1. The modification of KEAP1 at the K39 residue by SUMO1 disrupts its control of NRF2. This disruption promotes the antioxidant activity of NRF2 to protect cells from anoxic conditions. This is shown to be physiologically relevant in a cell line-derived xenograft model of tumor growth.

## Discussion

The post-translational modification of NRF2, including phosphorylation, ubiquitination, acetylation, and SUMOylation affect its subcellular location and activity. Here we recognize that the SUMOylation of its major regulator KEAP1 alternatively controls NRF2 activity. We identify that the lysine residue at position 39 on KEAP1 is modified by SUMO1. Without altering the stability, dimerization or cellular location of KEAP1 the SUMOylation of KEAP1 disrupted the protein control of NRF2. This manifested as reduced levels of NRF2 ubiquitination, apparently as a result of changes in the formation of the NRF2 ubiquitin complex. Accordingly, the SUMOylation of KEAP1 led to the accumulation of NRF2 and increased expression of NRF2-regulated genes. As a result, the SUMOylation of KEAP1 promoted the growth of H1299 lung cancer cells to promote tumor progression in this study. This is the first study showing that K39 is a SUMO1-targeted lysine site on KEAP1 that facilities NRF2 accumulation and functions.

SUMOylation has been shown to affect protein–protein interactions and regulate the nuclear localization of proteins (26) or their ubiquitination and subsequent degradation (27). Our study showed that KEAP1 SUMOylation was not related to the protein's nuclear localization or stability and didn’t affect its association with NRF2 or CUL3. However, the modification of KEAP1 by SUMO1 disrupted the copurification of NRF2 and CUL3. Our current understanding of the NRF2 ubiquitin complex doesn’t suggest a direct interaction between CUL3 and NRF2. Besides being bridged by KEAP1, these proteins are connected by the Ring-box 1 (RBX1) and ubiquitin-bound E2 proteins that constitute this ubiquitin ligase. RBX1 directly binds to CUL3 and recruits the E2 protein, which associates with NRF2 (28). Accordingly, our data appear to recognize that the SUMOylation of KEAP1 disrupts the recruitment of RBX1 by CUL3 and/or, less evidently, the recruitment of the E2 enzyme to RBX1 or reduces the interaction between the E2 enzyme and NRF2. Identification of which of these scenarios pertains to the SUMOylated KEAP1 would contribute to our understanding of the formation of NRF2 ubiquitin ligase and its function. This knowledge may assist the targeting of this pathway for chemoprevention or chemotherapy by controlling the NRF2-dependent redox response.

NRF2 is the main transcription factor of the intracellular antioxidant response. Drug resistance to chemotherapeutics is mediated by the overactivation of NRF2 and upregulated antioxidant genes, which impacts the function of drug efflux pumps and drug-metabolizing enzymes (29). It has been reported that tumor growth in lung, prostate, and gallbladder cancers was inhibited by suppressing NRF2 expression. This has also been reported to increase the sensitivity to various anticancer drugs. Our data identify KEAP1 as a potential tumor suppressor through its control of NRF2. We identify that SUMOylation of KEAP1 promotes NRF2 activity, thereby identifying a novel strategy to control NRF2. The activity that we have described recognizes the potential benefit of inhibiting the SUMOylation of KEAP1 as a cancer therapy.

In conclusion, our study discovered that KEAP1 is SUMOylated and demonstrated that this modification altered its regulation of NRF2 by suppressing its ubiquitin-dependent degradation. As a result of the ensuing accumulation of NRF2, the expression of NRF2-regulated antioxidant genes becomes elevated. This promotes cell tolerance to oxidative stress and increased the growth of tumors. These results revealed a new mechanism that controls the CUL3 E3 ligase by its adaptor protein KEAP1 and recognizes that inhibiting the SUMOylation of KEAP1 may be a promising strategy for tumor therapy.

## Experimental procedures

### Cell culture and transfection

HEK-293T and H1299 were purchased from the American Type Culture Collection (ATCC, Manassas, VA, USA). *KEAP1*-knockdown H1299 stabilized cell lines, KEAP1-overexpressed H1299 stabilized cell lines, and KEAP1-K39R overexpressed H1299 stabilized cell lines were constructed by our laboratory. Mutants of KEAP1 were generated by PCR-mediated, site-directed mutagenesis. PCR primers for gene cloning and site-directed mutagenesis are listed in Table S1. HEK-293T was cultured using Dulbecco's Modified Eagle Medium (Meilumbio) containing 10% (v/v) fetal bovine serum (FBS, ExCell Bio) and 100 μg/ml penicillin-streptomycin (Meilumbio). H1299 cell lines were cultured with Roswell Park Memorial Institute (RPMI) 1640 (Meilumbio) supplemented with 10% (v/v) FBS and 100 μg/ml of penicillin-streptomycin. Cells were cultured in a 37 °C incubator containing 5% CO_2._ . Transfected used the EZ Trans reagent (Shanghai Life iLab Bio Technology Co. Ltd) following the manufacturer’s instructions.

### Cellular treatments

To inhibit SUMOylation, cells were cultured with 100 μM of protein SUMOylation inhibitor 2-D08 (Selleck) for 12 h. To inhibit proteasome function, cells were cultured with 20 μM MG132 (MCE) for the indicated times. To inhibit protein synthesis, cells were cultured with 100 ug/ml cycloheximide (Meilumbio) for the indicated times.

### Western blotting

The cells culture dish was placed on ice before being washed with pre-chilled PBS, lysed in SDS buffer on ice, and boiled for 10 min at 100 °C. Then, sample proteins were loaded onto SDS-PAGE gels, followed by transfer to polyvinylidene fluoride membranes (Millipore). After blocking in 5% (w/v) skim milk powder to block non-specific binding sites, the membrane containing proteins was incubated with primary antibodies (HA, CST, 1:1000; KEAP1, Proteintech, 1:1000; NRF2, Proteintech, 1:100; actin, CST, 1:3000) overnight followed by secondary antibodies (goat anti-mouse IgG horseradish peroxidase (HRP), CST, 1:5000; goat anti-rabbit IgG HRP, 1:5000, Beyotime) for 1 h at room temperature, then imaged through a Tanon 4500 Chemiluminescence Imager using a previously described method (30). Full blots of images cropped for presentation are presented in the supporting information (Figure 1, Figure 2, Figure 3, Figure 4, Figure 5).

### Immunoprecipitation

Cells were washed in pre-cooled PBS and lysed in an IP lysis buffer (Beyotime) supplemented with protease inhibitor cocktail tablets (Roche) for 30 min, then the lysates were centrifuged at 12,000*g* for 10 min and 10% (v/v) of the supernatant was collected as the input. The remaining lysates were incubated with the indicated antibodies, rabbit control IgG (Diagenode) or anti-Myc antibody (CST) with the addition of protein A/G beads (Millipore) or incubated with anti-HA beads (EveryLab) alone overnight at 4 °C with slow shaking. The immunoprecipitants were washed three times with pre-cooled IP wash buffer (20 mM tris-HCl, 5 mM EDTA, 150 mM NaCl and 0.05% (v/v) Nonidet-P40), then 2 × SDS loading buffer was added and the samples were boiled for 5 min, followed by WB analysis with the antibodies of interest (HA, CST, 1:1000; Myc, CST, 1:1000; FLAG, 1:1000, CST; NRF2, Proteintech, 1:1000; actin, CST, 1:3000) using a previously described method (31).

### *In vivo* SUMOylation and ubiquitination assay

For SUMOylation assays under denaturing conditions, HEK293T cells transfected with various plasmids were lysed in IP lysis buffer (8 M urea, 50 mM Na2HPO4/NaH2PO4 (pH 7.4), 300 mM NaCl and 0.1% TritonX-100) containing protease inhibitors and 20 mM imidazole. The cell lysates were incubated with Ni Ultraflow resin (Sangon Biotech) overnight with gentle agitation at 4 °C. The resin was then washed five times at room temperature then His-tagged proteins were eluted in elution buffer and the immunoprecipitants were subjected to WB with the indicated antibodies (HA, CST, 1:1000; FLAG, CST, 1:1000; His,1:1000, CST; Actin, CST, 1:3000, using a previously described method (14).

For ubiquitination and SUMOylation assays under denaturing conditions, cells were lysed in IP lysis buffer containing protease inhibitors and 1% (v/v) SDS by rigorous scraping on ice for 30 min and then boiled for 5 min to denature the proteins. Then the lysates were diluted 10 times with lysis buffer without SDS and incubated with the indicated antibody rabbit control IgG or the anti-NRF2 antibody (Proteintech) and the addition of protein A/G beads or incubated with anti-HA beads overnight at 4 °C with slowly shaking. The immunoprecipitants were washed three times with pre-cooled IP wash buffer and then analyzed by WB with the antibodies of interest (HA, CST, 1:1000; Myc, CST, 1:1000; KEAP1, Proteintech, 1:1000; NRF2, Proteintech,1:1000; Ub, CST,1:1000; SUMO1, CST, 1:1000; actin, CST, 1:3000) using a previously described method (32, 33).

### Protein association assay

WT and mutant KEAP1 was expressed in HEK-293T cells as HA-tagged constructs with FLAG-Keap1) then immunoprecipitated with anti-FLAG antibody (MBL). The immunoprecipitants were washed three times with pre-cooled IP wash buffer and then analyzed by WB with the antibodies of interest (KEAP1, Proteintech, 1:1000; Flag, CST, 1:3000).

### Immunofluorescence staining

H1299 cell lines were seeded on round glass coverslips in 24-well plates overnight. After washing with PBS twice, cells were fixed in 4% paraformaldehyde at room temperature for 30 min, permeabilized in 0.2% TritonX-100 for 30 min, then blocked with 10% goat serum in PBS for 1 h. Cells were incubated with primary antibody (KEAP1, Proteintech, 1:500) overnight at 4 °C. The next day, after washing in PBS three times, cells were incubated with a fluorescent secondary antibody (DyLight 594, Invitrogen, 1:1000) at room temperature for 1 h in the dark, washed three times with PBS and counterstained with DAPI for 5 min. Slides were mounted with nail polish and imaged by an ECLIPSE Ti microscope (Nikon). Images were analyzed with ImageJ software as described previously (31).

### Measurement of the half-life of KEAP1

HEK-293T cells were transfected with overexpression plasmids using EZ Trans for 48 h and treated with cycloheximide (100 μg/ml) at different time points up to 16 h. Cells were washed once in PBS and lysed with SDS buffer. Total cell lysates were electrophoresed using SDS-PAGE followed by WB analysis, as described previously (32).

### Assay of luciferase reporter gene expression

HEK-293T cells were transfected with 100 ng of pARE-Luc, 10 ng of pRL-TK and ng of NRF2 plasmids with either the KEAP1-WT or KEAP1-K39R constructs. The activity of firefly and Renilla luciferase was measured by Dual Luciferase Reporter Gene assay (Beyotime). Firefly luciferase activity was normalized to Renilla luciferase activity to control sample-to-sample differences, as previously described (34).

### RNA extraction and real-time PCR

To quantitatively analyze gene expression, total RNA was extracted from cells or tumor tissue using TRIZOL reagent (Ambion) following the manufacturer’s instructions. The RNA was treated with DNase and the concentration was measured by a NANO-100 Micro Spectrophotometer (ALLSHENG). Complementary DNA was synthesized using a PrimeScript RT regent kit with gDNA Eraser (TaKaRa) with 1 μg of total RNA following the manufacturer’s protocols. Quantitative real-time PCR (qRT-PCR) was performed using TB Green Premix Ex Taq II (TaKaRa) with a QuantStudio 6 Pro Real-Time PCR system (Thermo Fisher Scientific). The data were expressed as relative mRNA levels and normalized to the *18S* rRNA as previously described (35). The sequence of the primers is shown in Table S2.

### ChIP assay

Cells (2 × 10^7^) were cross-linked then nuclear lysates were prepared using a Sonication ChIP Kit (ABclonal) to shear DNA to approximately 500 bp. The samples were immunoprecipitated overnight at 4 °C using IgG or anti-NRF2 antibody (Proteintech) and the levels of target genes were determined *via* RT-qPCR. The primers used in this study are listed in Table S3.

### Cell Counting Kit-8 assays

Cell viability was analyzed by a Cell Counting Kit-8 assay (CCK8, Beyotime), as previously described (36). H1299 stable cell lines were seeded at a density of 2 × 10^3^/well in 100 μl of medium on 96-well plates (Corning). After culturing for 0, 1, 2, 3, 4, 5, or 6 days 10 μl of CCK8 reagent was added and cells were incubated for 2 h. The absorbance at 450 nm was measured with a SpetraMax M3 microplate reader (Molecular Devices). The wells without cells were used as blanks.

### Reactive oxygen species assay

To measure cellular ROS levels, the cells were washed in PBS and incubated with 10 μM of dichloro-dihydro-fluorescein diacetate (DCFH-DA) (Beyotime) at 37 °C for 30 min. Cells were treated with Rosup as a positive control. Treated cells were washed in PBS, trypsinized and collected in pre-cooled PBS then analyzed on a BD FACSCanto II flow cytometry system (BD Biosciences). The data were analyzed using FlowJo V10 software, as previously described (37).

### Immunohistochemistry

Tumor tissues were dissected from individual mice then immediately fixed with 4% paraformaldehyde and embedded in paraffin as described previously (35). The embedded sections were sliced into 4 μ sections for immunostaining with anti-Ki67 antibodies (CST) at 4 °C overnight and then incubated with HRP-conjugated secondary antibody at 37 °C for 2 h. The sections were counterstained with hematoxylin. At least three images of randomly selected microscopic fields were captured from each slide from each mouse.

### Tumor xenograft assay

Five-week-old BALB/c nude mice (five per group) were subcutaneously injected with 2.5 × 10^6^ of H1299-shKEAP1-WT or H1299-shKEAP1-K39R cells. The tumor size was measured every 3 days and the experiment ended when the tumor reached approximately 1 cm in at least one dimension, at which point the mice were euthanized and the tumors were dissected. Tumor volume was calculated according to the following equation: length × width × 0.5 × width. All animal procedures were approved by the Shanghai Jiao Tong University Medical Animal Ethics Committee.

### Statistical analysis

Data is represented as mean ± sd. Unpaired Student’s *t*-tests were used to compare the differences between two groups, and one-way ANOVA was used for comparisons among multiple groups. All data was analyzed using Graph Prism 8.0 software (GraphPad Software Inc., San Diego, CA, USA). A *p* value < 0.05 was considered statistically significant.

## Data availability

The data that support the ﬁndings of this study are available from the corresponding author upon reasonable request.

## Supporting information

This article contains supporting information. All primers used in this study are listed in Supplementary Tables 1, 2 and 3.

## Conflict of interest

The authors declare that the research was conducted in the absence of any commercial or financial relationships that could be construed as a potential conflict of interest.
