# Supplementary material for: SUMOylation of the ubiquitin ligase component KEAP1 at K39 upregulates NRF2 and its target function in lung cancer cell proliferation
Source: J Biol Chem. 2023 Sep 1;299(10):105215. doi: 10.1016/j.jbc.2023.105215 (PMC10556770; doi:10.1016/j.jbc.2023.105215)
Supplement: Supporting information [file mmc1.pdf]

Full immunoblots

Figure1

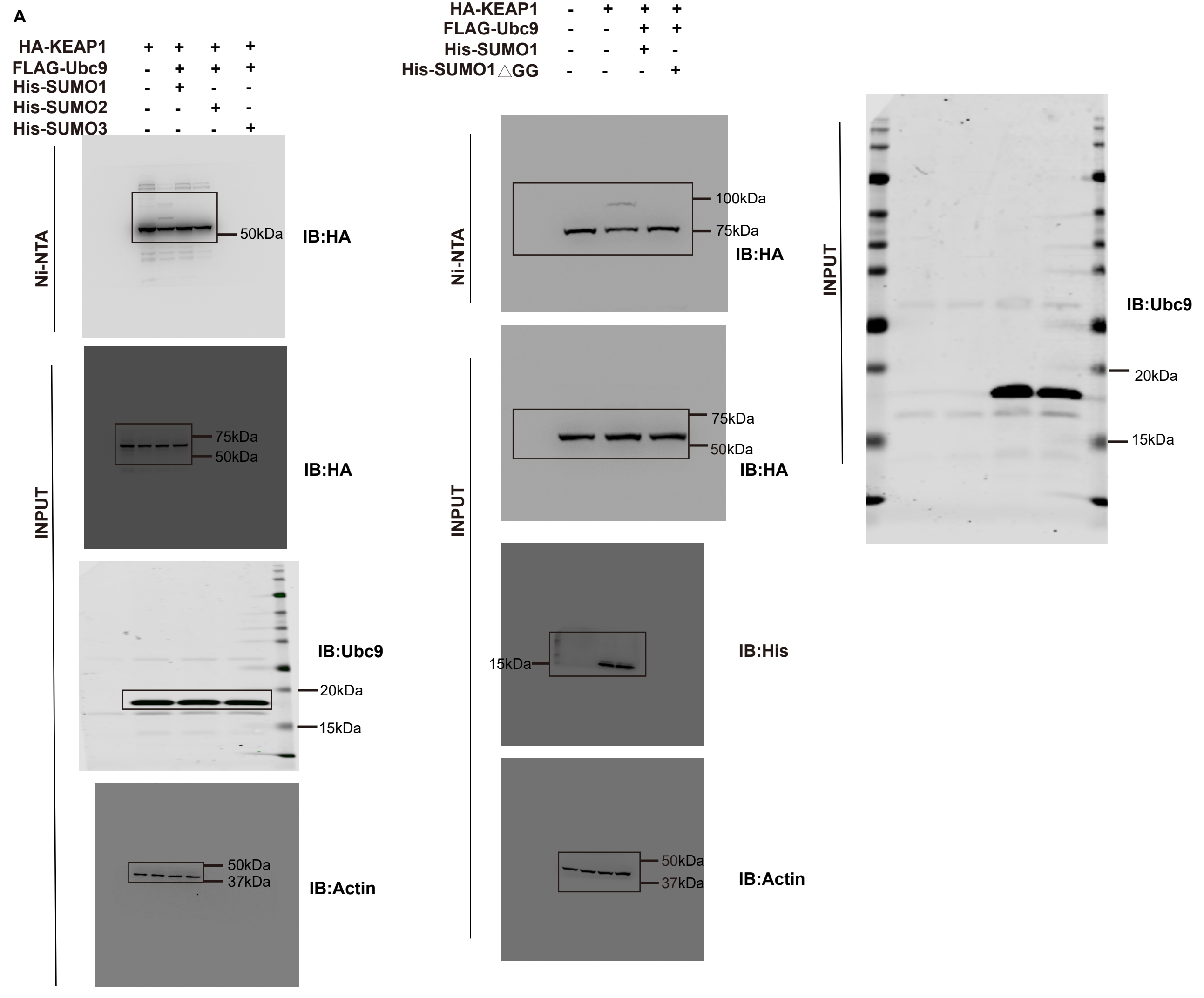

Figure1

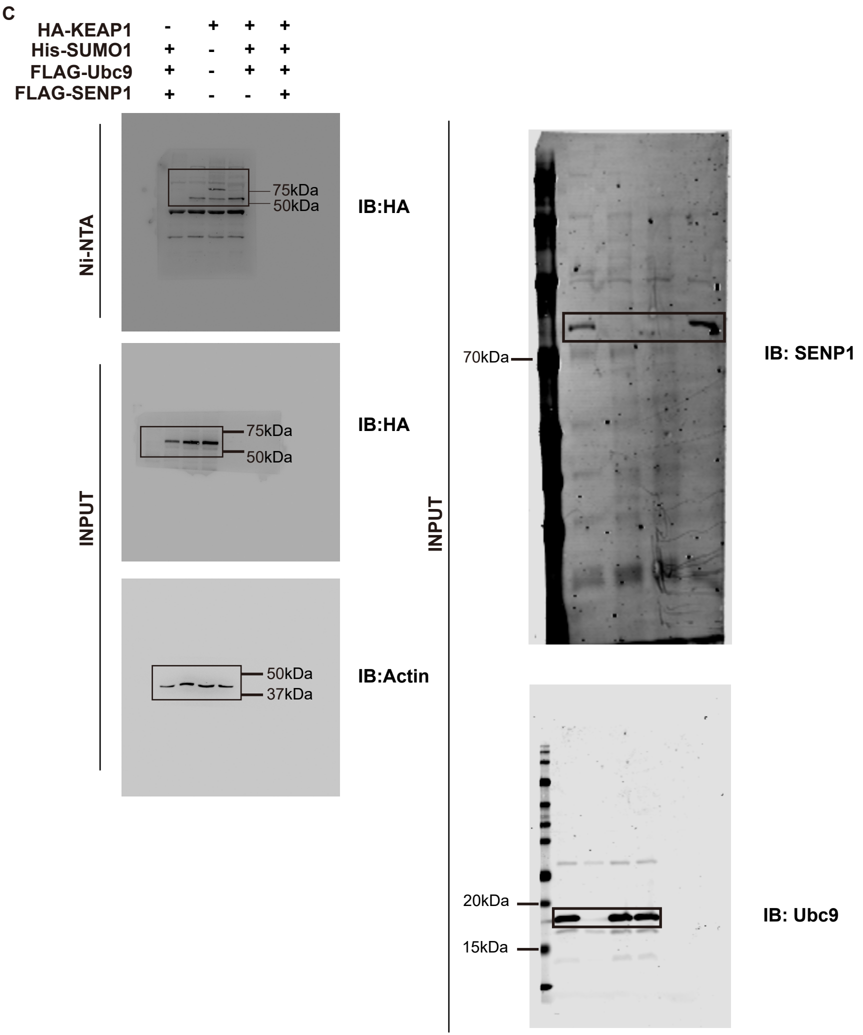
C
HA-KEAP1 - + + +
His-SUMO1 + - + +
FLAG-Ubc9 + - + +
FLAG-SENP1 + - - +
Ni-NTA
75kDa
50kDa
IB:HA
INPUT
75kDa
50kDa
IB:HA
50kDa
37kDa
IB:Actin
INPUT
70kDa
IB: SENP1
20kDa
15kDa
IB: Ubc9

Figure 1

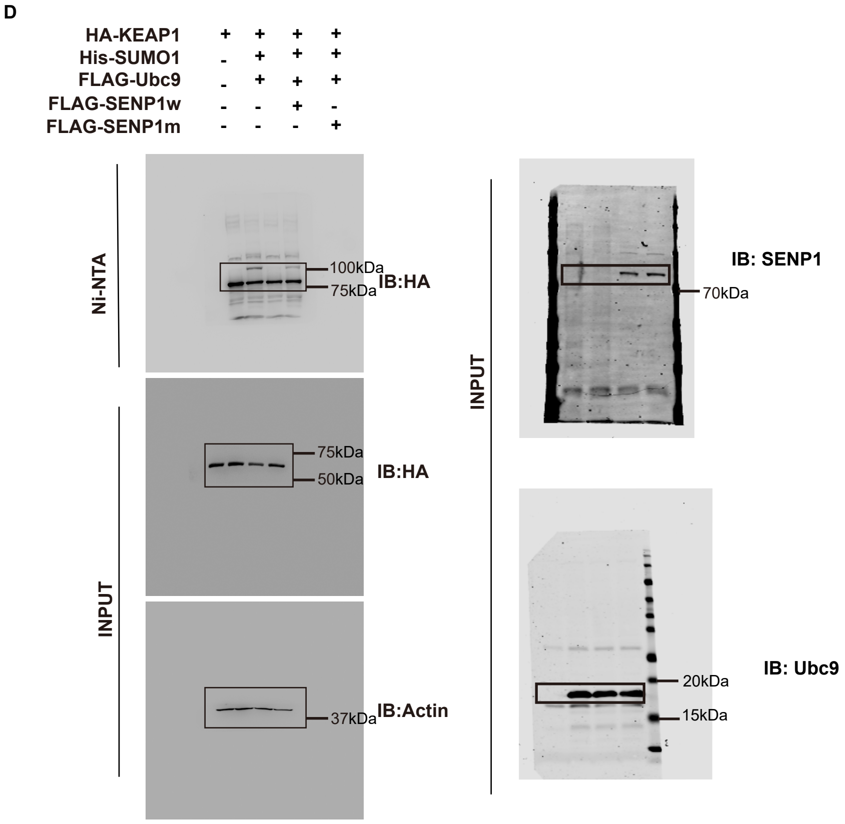
D
HA-KEAP1 + + + +
His-SUMO1 - + + +
FLAG-Ubc9 - + + +
FLAG-SENP1w - - + -
FLAG-SENP1m - - - +
Ni-NTA
100kDa
75kDa
IB:HA
INPUT
75kDa
50kDa
IB:HA
37kDa
IB:Actin
INPUT
IB: SENP1
70kDa
IB: Ubc9
20kDa
15kDa

Figure 1

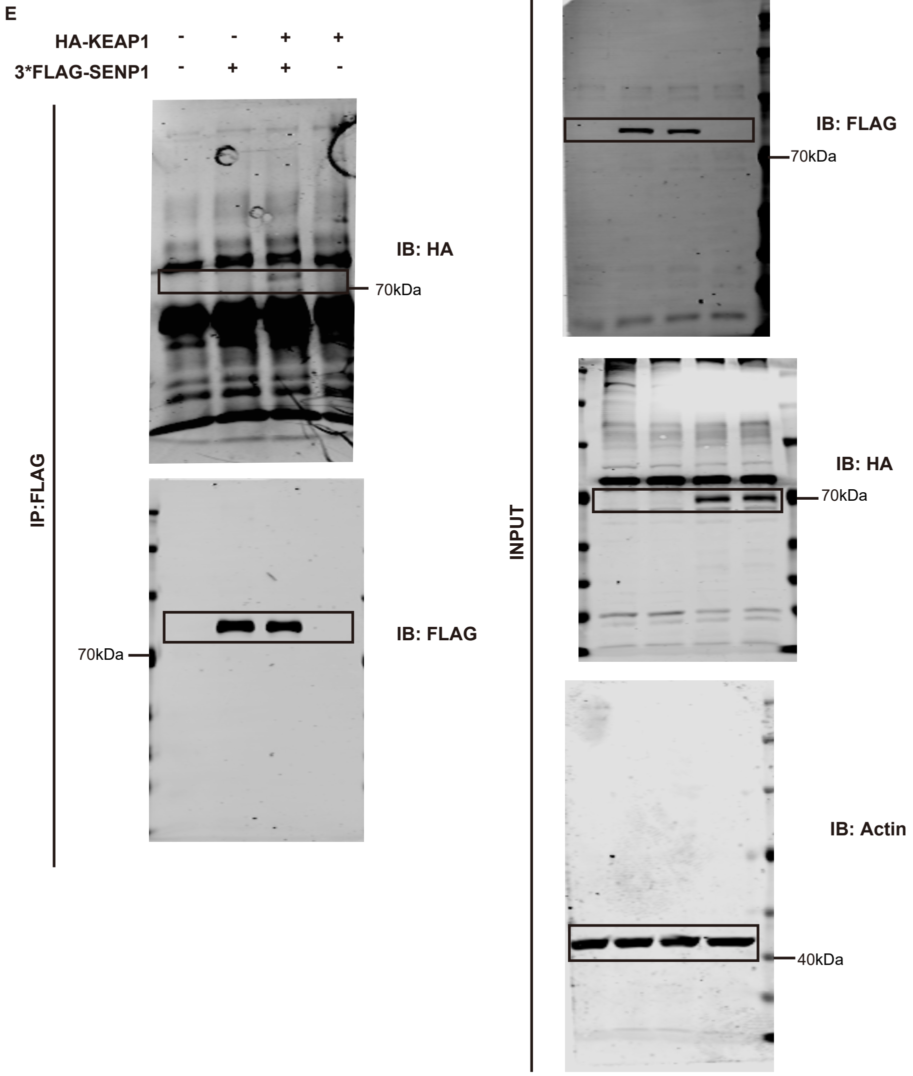
E
HA-KEAP1 - - + +
3*FLAG-SENP1 - + + -
IP:FLAG
IB: HA
70kDa
IB: FLAG
70kDa
INPUT
IB: FLAG
70kDa
IB: HA
70kDa
IB: Actin
40kDa

Figure 1

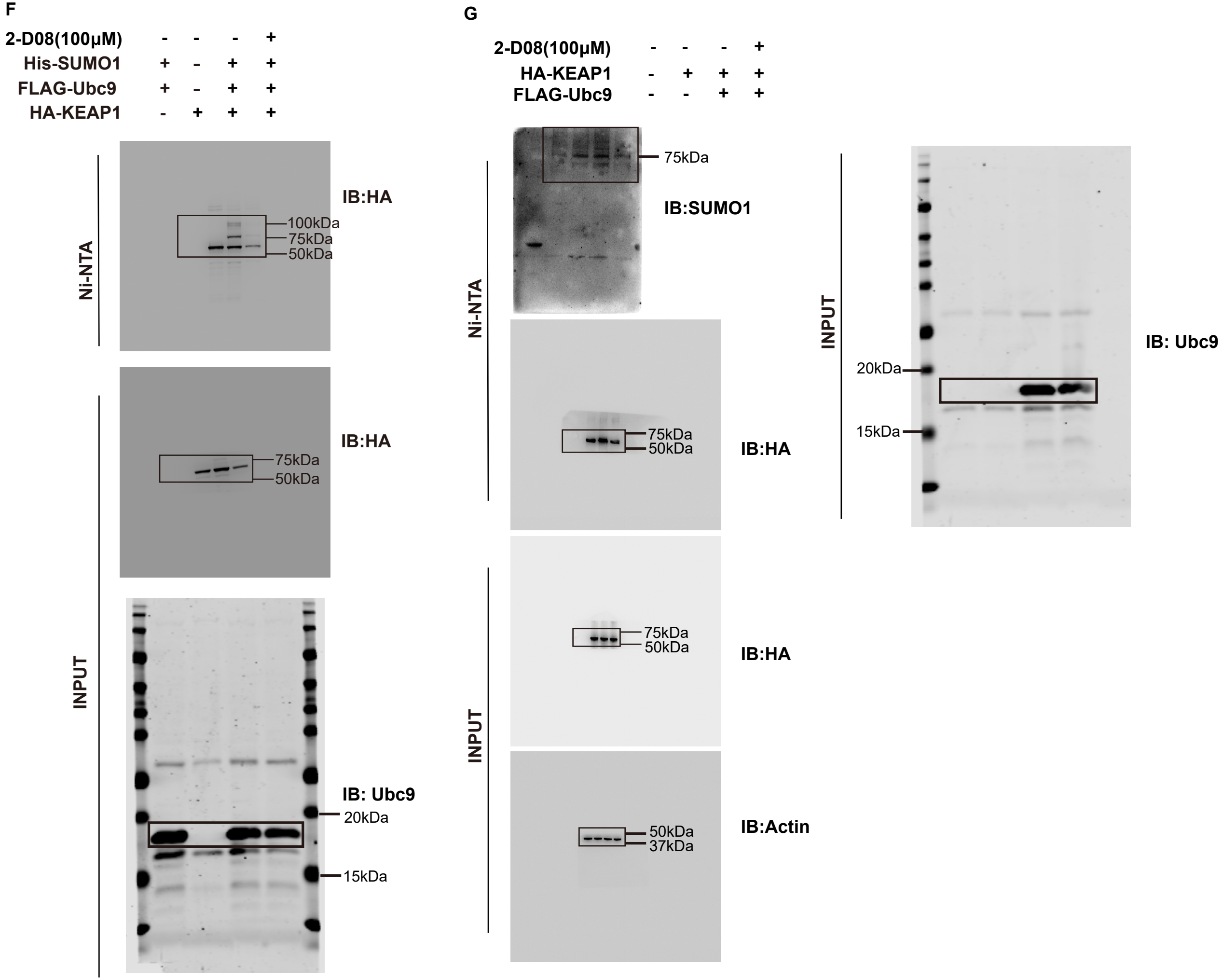
F
2-D08(100μM) - - - +
His-SUMO1 + - + +
FLAG-Ubc9 + - + +
HA-KEAP1 - + + +
Ni-NTA
IB:HA
100kDa
75kDa
50kDa
INPUT
IB:HA
75kDa
50kDa
IB: Ubc9
20kDa
15kDa
G
2-D08(100μM) - - - +
HA-KEAP1 - + + +
FLAG-Ubc9 - - + +
Ni-NTA
75kDa
IB:SUMO1
IB:HA
75kDa
50kDa
INPUT
IB:HA
75kDa
50kDa
IB:Actin
50kDa
37kDa
INPUT
IB: Ubc9
20kDa
15kDa

Figure 2

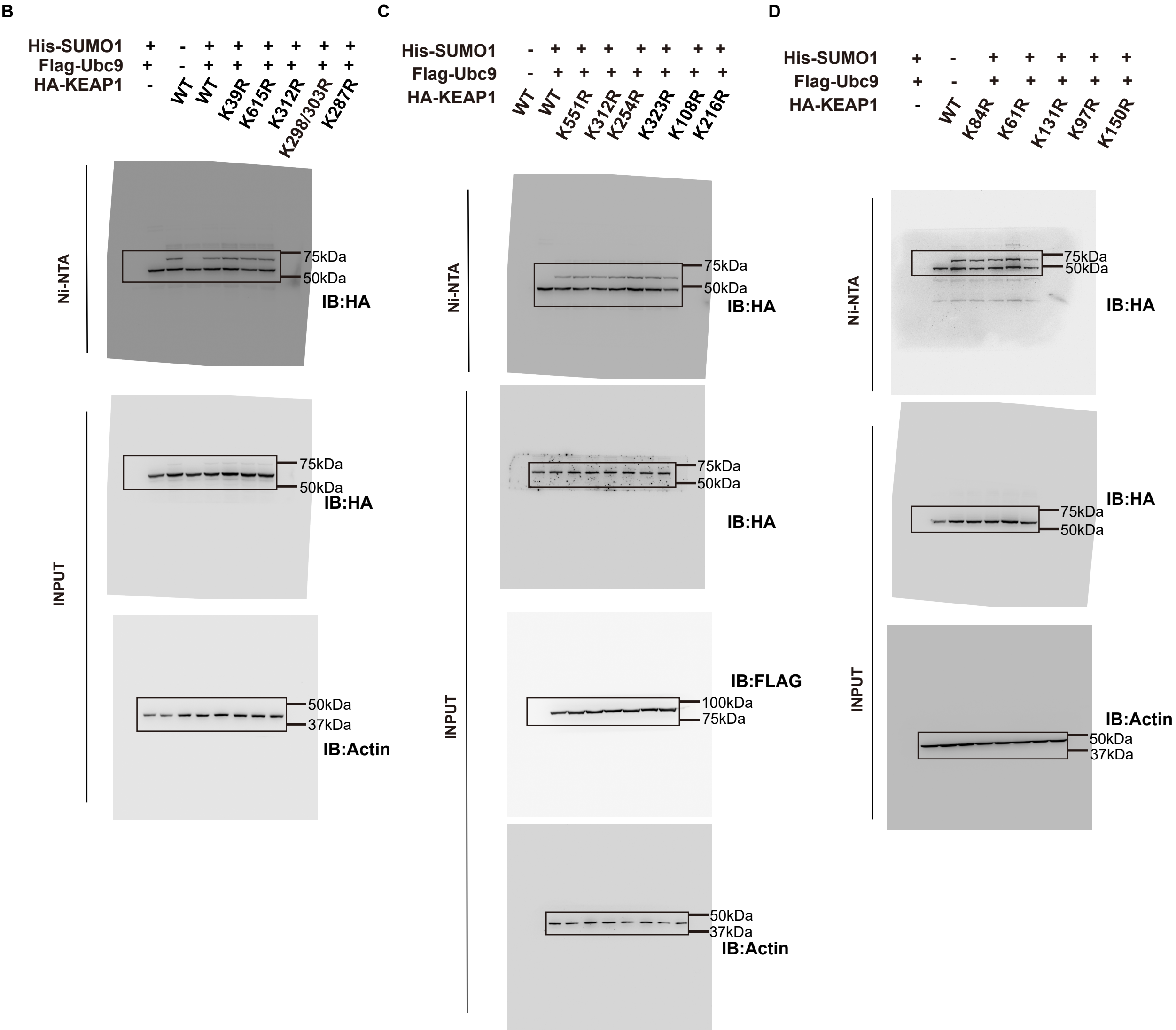
B
His-SUMO1
Flag-Ubc9
HA-KEAP1
WT
WT
K39R
K615R
K312R
K298/303R
K287R
Ni-NTA
75kDa
50kDa
IB:HA
INPUT
75kDa
50kDa
IB:HA
50kDa
37kDa
IB:Actin
C
His-SUMO1
Flag-Ubc9
HA-KEAP1
WT
WT
K551R
K312R
K254R
K323R
K108R
K216R
Ni-NTA
75kDa
50kDa
IB:HA
INPUT
75kDa
50kDa
IB:HA
IB:FLAG
100kDa
75kDa
50kDa
37kDa
IB:Actin
D
His-SUMO1
Flag-Ubc9
HA-KEAP1
WT
K84R
K61R
K131R
K97R
K150R
Ni-NTA
75kDa
50kDa
IB:HA
INPUT
IB:HA
75kDa
50kDa
IB:Actin
50kDa
37kDa

Figure 2

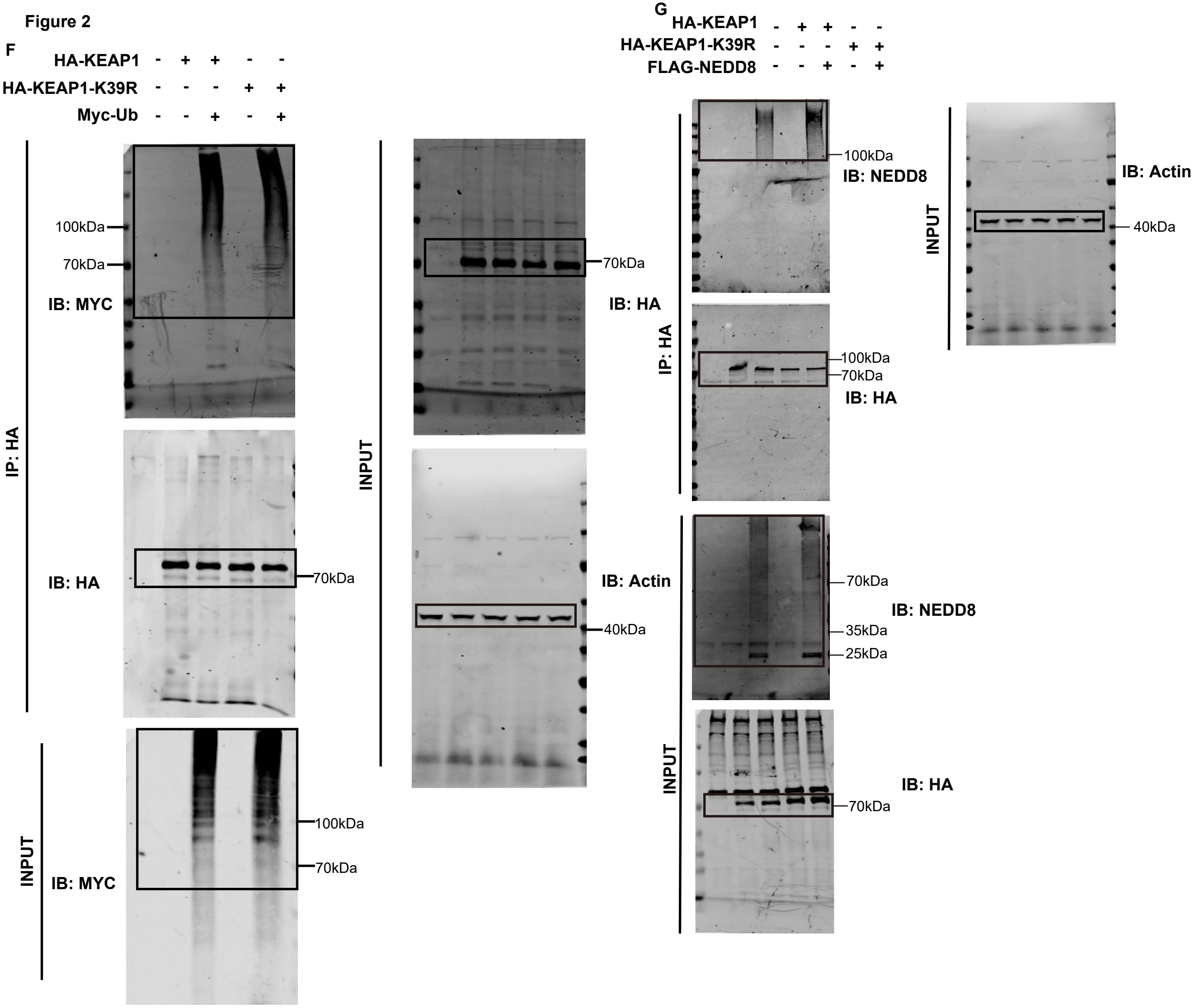

F
HA-KEAP1 - + + - -
HA-KEAP1-K39R - - - + +
Myc-Ub - - + - +
IP: HA
100kDa
70kDa
IB: MYC
IB: HA
70kDa
INPUT
IB: MYC
100kDa
70kDa
INPUT
70kDa
IB: HA
IB: Actin
40kDa
G
HA-KEAP1 - + + - -
HA-KEAP1-K39R - - - + +
FLAG-NEDD8 - - + - +
100kDa
IB: NEDD8
IP: HA
100kDa
70kDa
IB: HA
INPUT
IB: Actin
40kDa
70kDa
IB: NEDD8
35kDa
25kDa
INPUT
IB: HA
70kDa

Figure 3

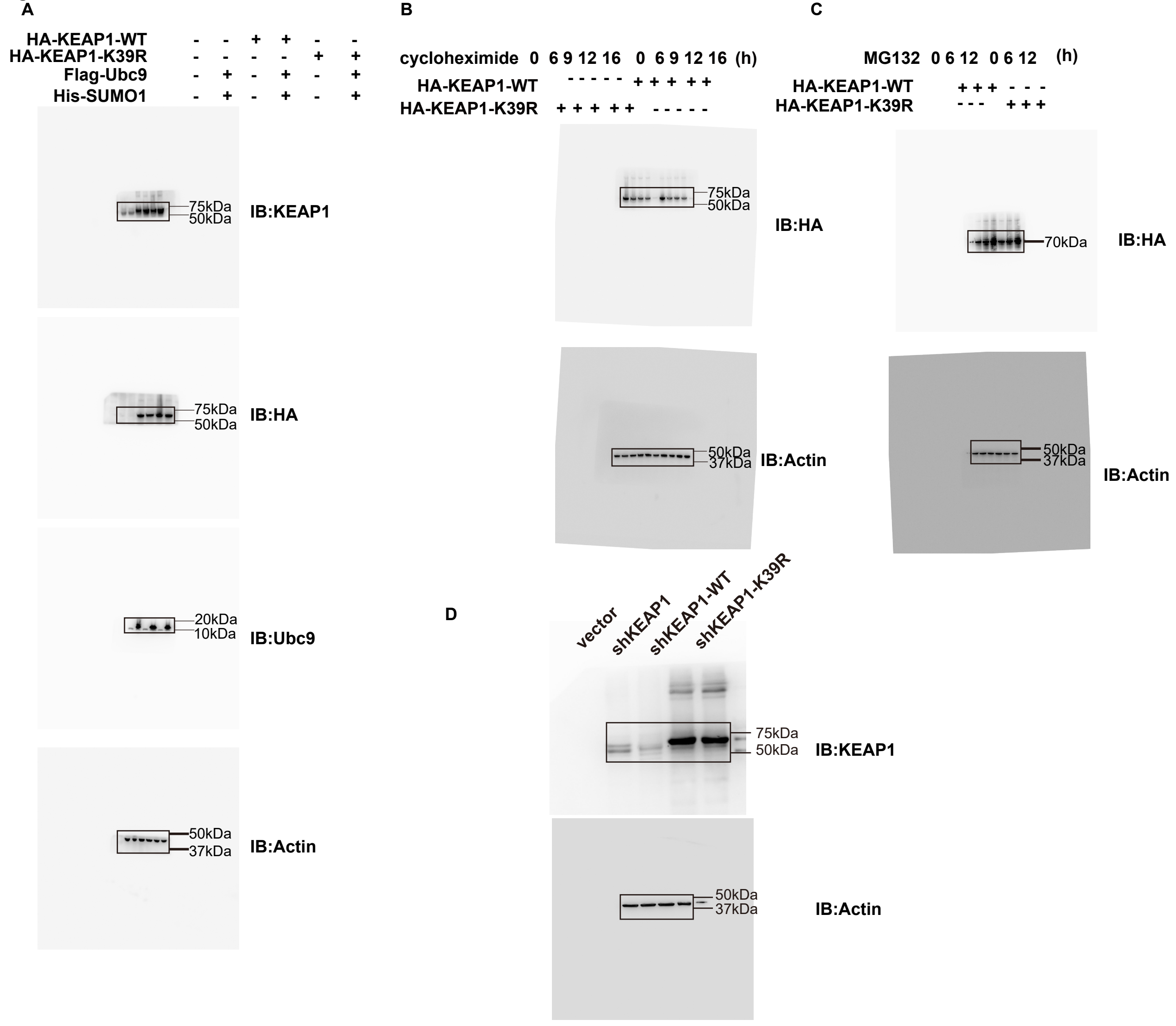
A
HA-KEAP1-WT - - + + - -
HA-KEAP1-K39R - - - - + +
Flag-Ubc9 - + - + - +
His-SUMO1 - + - + - +
75kDa
50kDa
IB:KEAP1
75kDa
50kDa
IB:HA
20kDa
10kDa
IB:Ubc9
50kDa
37kDa
IB:Actin
B
cycloheximide 0 6 9 12 16 0 6 9 12 16 (h)
HA-KEAP1-WT - - - - - + + + + +
HA-KEAP1-K39R + + + + + - - - - -
75kDa
50kDa
IB:HA
50kDa
37kDa
IB:Actin
C
MG132 0 6 12 0 6 12 (h)
HA-KEAP1-WT + + + - - -
HA-KEAP1-K39R - - - + + +
70kDa
IB:HA
50kDa
37kDa
IB:Actin
D
vector
shKEAP1
shKEAP1-WT
shKEAP1-K39R
75kDa
50kDa
IB:KEAP1
50kDa
37kDa
IB:Actin

Figure 3

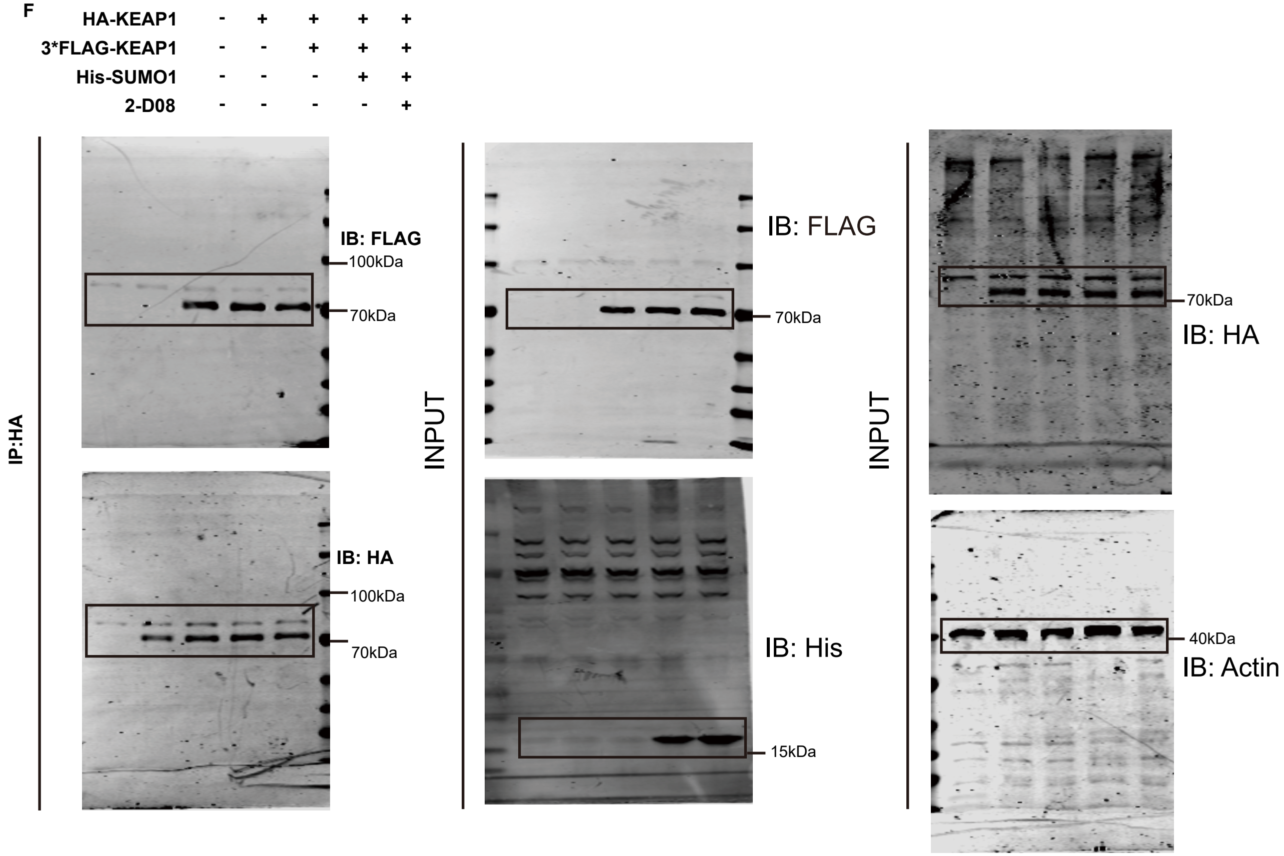
F
HA-KEAP1 - + + + +
3*FLAG-KEAP1 - - + + +
His-SUMO1 - - - + +
2-D08 - - - - +
IP:HA
IB: FLAG
100kDa
70kDa
IB: HA
100kDa
70kDa
INPUT
IB: FLAG
70kDa
IB: His
15kDa
INPUT
70kDa
IB: HA
40kDa
IB: Actin

Figure 3

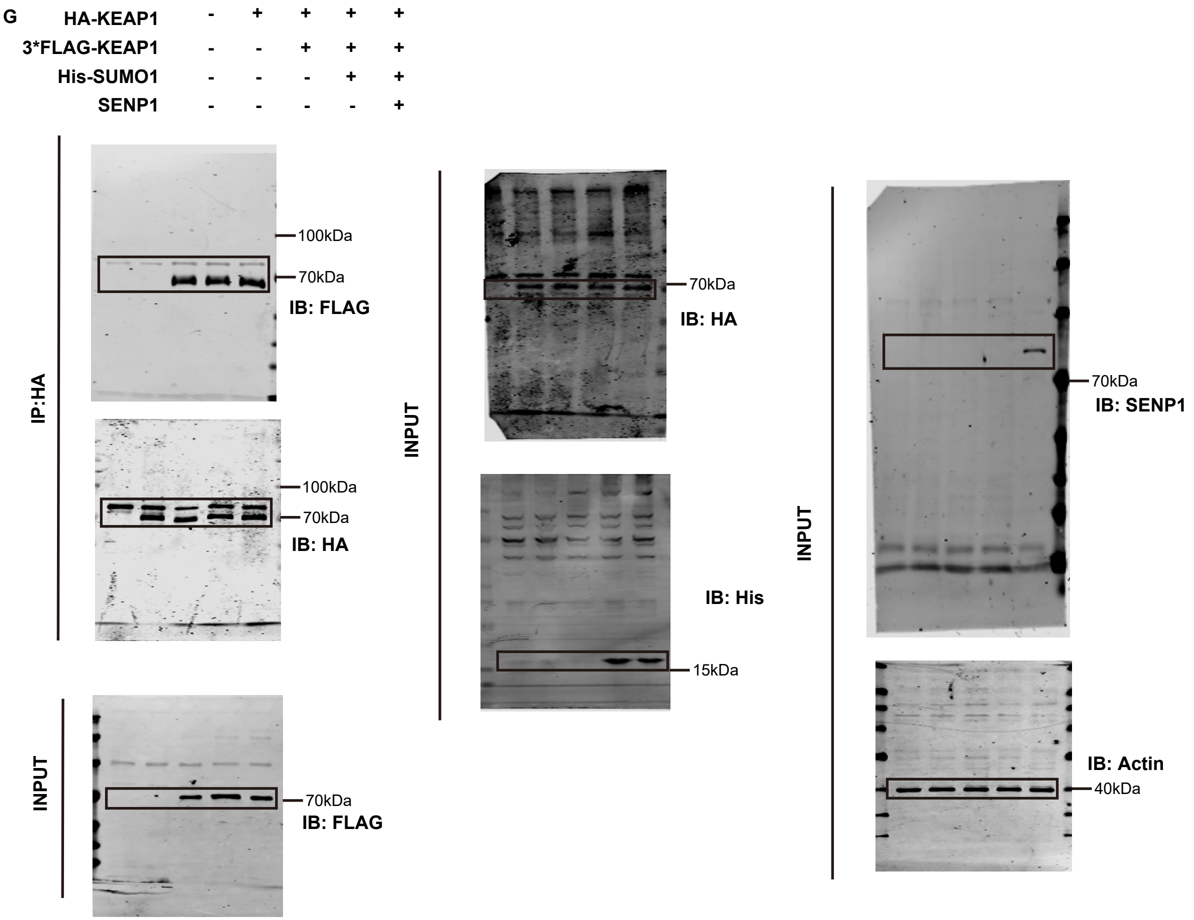

G
HA-KEAP1 - + + + +
3*FLAG-KEAP1 - - + + +
His-SUMO1 - - - + +
SENP1 - - - - +
IP:HA
100kDa
70kDa
IB: FLAG
100kDa
70kDa
IB: HA
INPUT
70kDa
IB: FLAG
INPUT
70kDa
IB: HA
IB: His
15kDa
INPUT
70kDa
IB: SENP1
IB: Actin
40kDa

Figure 3

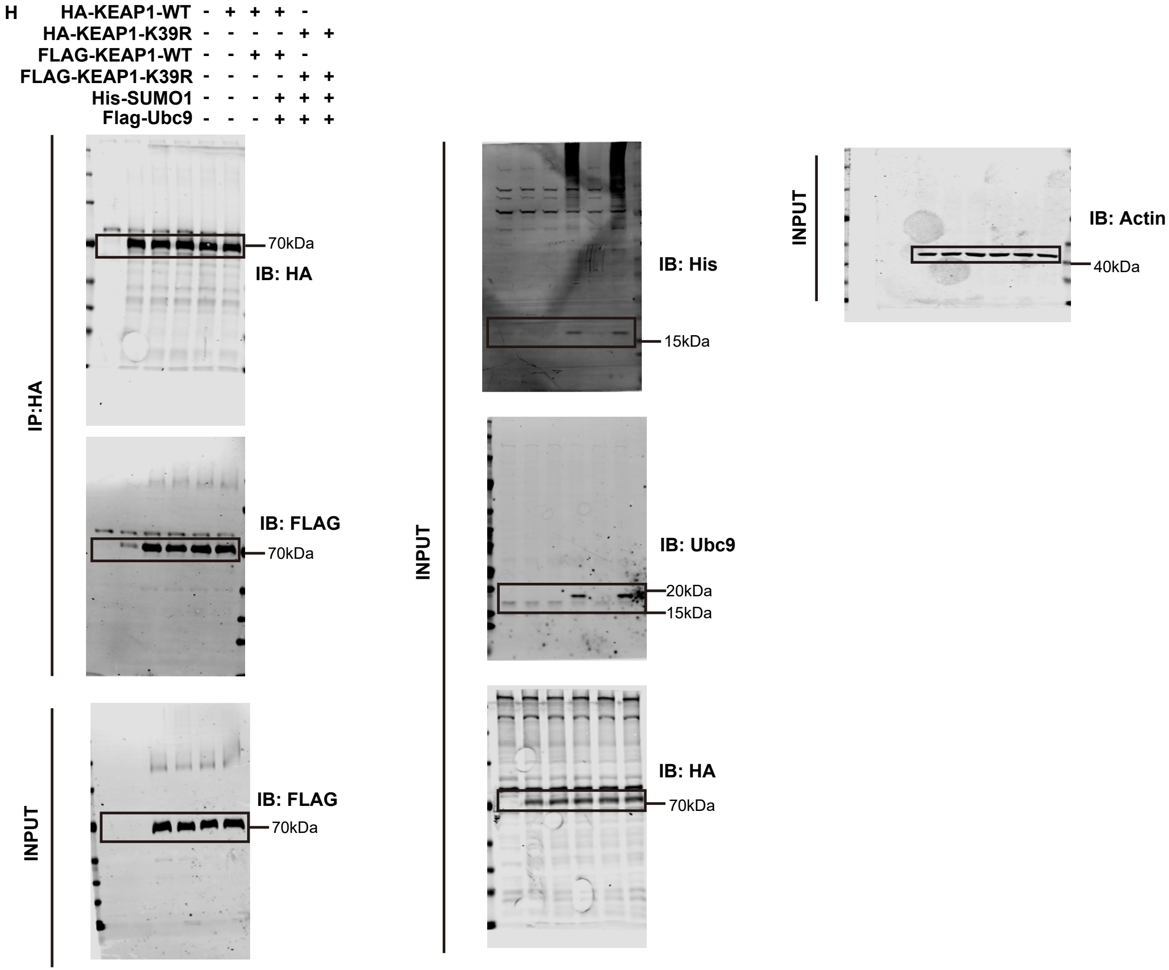
H
HA-KEAP1-WT - + + + -
HA-KEAP1-K39R - - - - + +
FLAG-KEAP1-WT - - + + -
FLAG-KEAP1-K39R - - - - + +
His-SUMO1 - - - + + +
Flag-Ubc9 - - - + + +
IP:HA
70kDa
IB: HA
IB: FLAG
70kDa
INPUT
IB: FLAG
70kDa
INPUT
IB: His
15kDa
IB: Ubc9
20kDa
15kDa
IB: HA
70kDa
INPUT
IB: Actin
40kDa

Figure 4

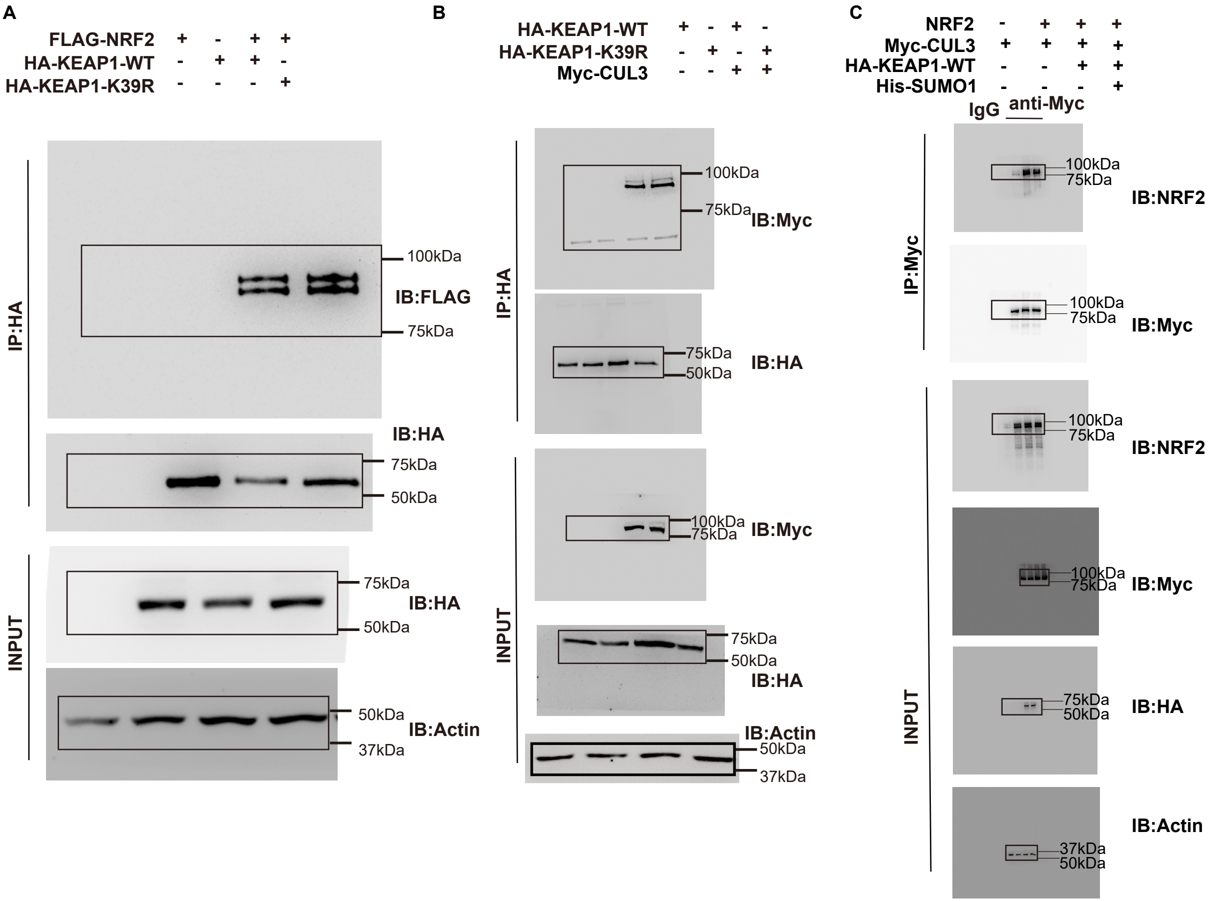
A
FLAG-NRF2 + - + +
HA-KEAP1-WT - + + -
HA-KEAP1-K39R - - - +
IP:HA
INPUT
100kDa
75kDa
IB:FLAG
IB:HA
75kDa
50kDa
IB:HA
75kDa
50kDa
IB:Actin
50kDa
37kDa
B
HA-KEAP1-WT + - + -
HA-KEAP1-K39R - + - +
Myc-CUL3 - - + +
IP:HA
INPUT
100kDa
75kDa
IB:Myc
IB:HA
75kDa
50kDa
IB:Myc
100kDa
75kDa
IB:HA
75kDa
50kDa
IB:Actin
50kDa
37kDa
C
NRF2 - + + +
Myc-CUL3 + + + +
HA-KEAP1-WT - - + +
His-SUMO1 - - - +
IgG
anti-Myc
IP:Myc
INPUT
100kDa
75kDa
IB:NRF2
100kDa
75kDa
IB:Myc
100kDa
75kDa
IB:NRF2
100kDa
75kDa
IB:Myc
75kDa
50kDa
IB:HA
37kDa
50kDa
IB:Actin

Figure 4

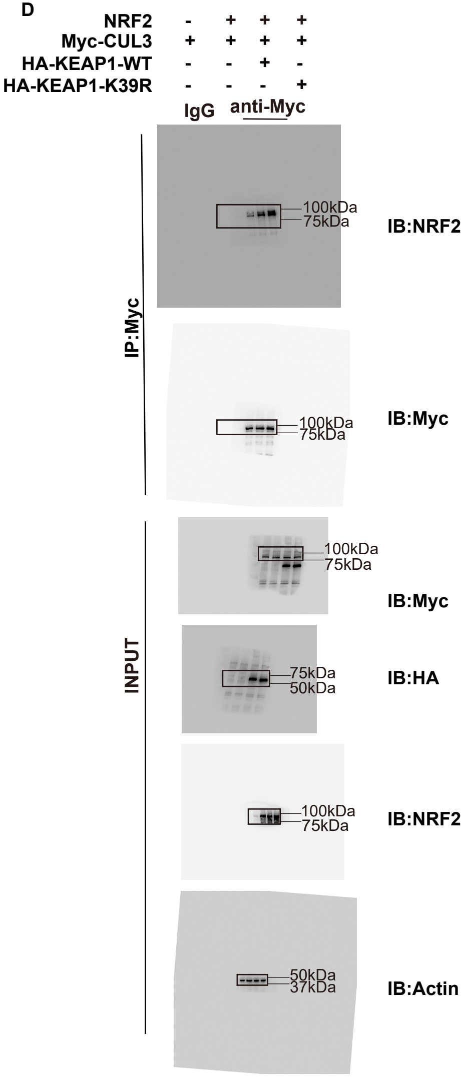
D
NRF2 - + + +
Myc-CUL3 + + + +
HA-KEAP1-WT - - + -
HA-KEAP1-K39R - - - +
IgG
anti-Myc
IP:Myc
100kDa
75kDa
IB:NRF2
100kDa
75kDa
IB:Myc
INPUT
100kDa
75kDa
IB:Myc
75kDa
50kDa
IB:HA
100kDa
75kDa
IB:NRF2
50kDa
37kDa
IB:Actin

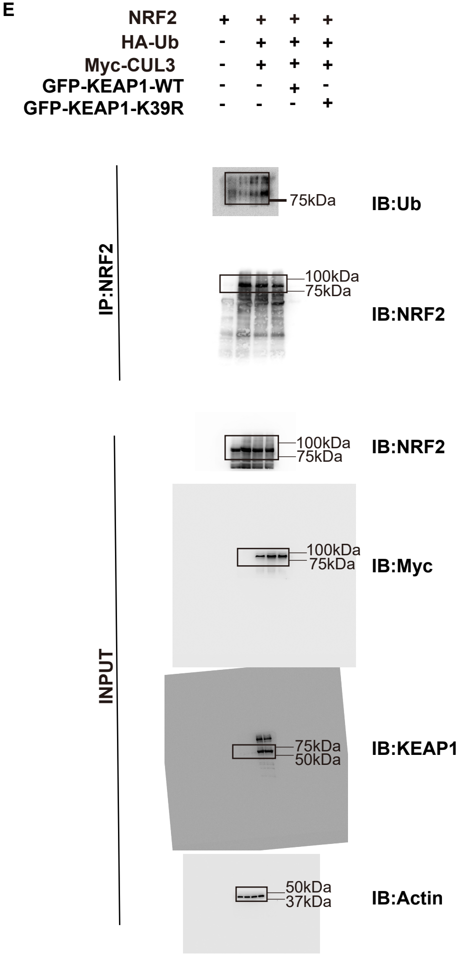
E
NRF2 + + + +
HA-Ub - + + +
Myc-CUL3 - + + +
GFP-KEAP1-WT - - + -
GFP-KEAP1-K39R - - - +
IP:NRF2
75kDa
IB:Ub
100kDa
75kDa
IB:NRF2
INPUT
100kDa
75kDa
IB:NRF2
100kDa
75kDa
IB:Myc
75kDa
50kDa
IB:KEAP1
50kDa
37kDa
IB:Actin

Figure 5

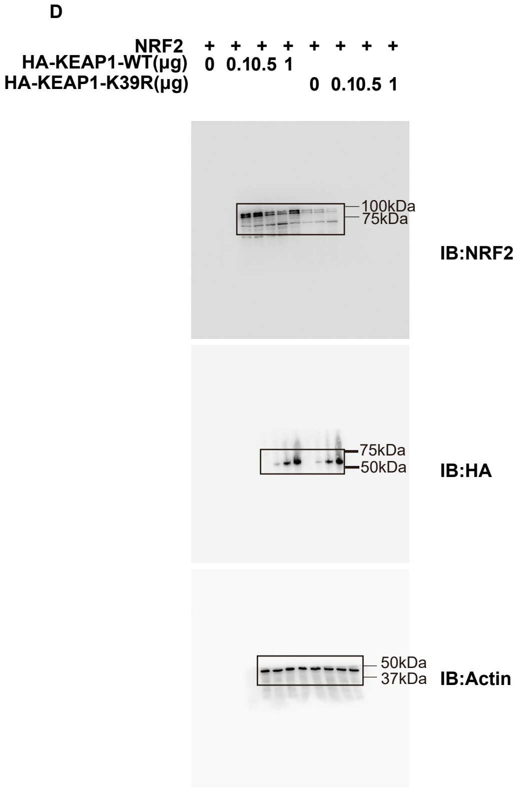
D
NRF2 + + + + + + + +
HA-KEAP1-WT(μg) 0 0.1 0.5 1
HA-KEAP1-K39R(μg) 0 0.1 0.5 1
100kDa
75kDa
IB:NRF2
75kDa
50kDa
IB:HA
50kDa
37kDa
IB:Actin
