# Supplementary material for: SUMOylation of the ubiquitin ligase component KEAP1 at K39 upregulates NRF2 and its target function in lung cancer cell proliferation
Source: J Biol Chem. 2023 Sep 1;299(10):105215. doi: 10.1016/j.jbc.2023.105215 (PMC10556770; doi:10.1016/j.jbc.2023.105215)
Supplement: Table S1 [file mmc2.docx]

Table S1 primers used for construction of KEAP1 mutants

| Primer | Sequence（5’-3’） |
| --- | --- |
| KEAP1_K39R_F | AGGGCGGAGGTGACGCCCTCC |
| KEAP1_K39R_R | GCACTCAGTGGAGGCGTACAT |
| KEAP1_K2616_F | AGGCAAGAGGAGTTCTTCAACCT |
| KEAP1_K2616_R | GGCCACCTCCCCAAAATGCATGT |
| KEAP1_K254R_F | AGGTACGACTGCGAACAGCGA |
| KEAP1_K254R_R | GACCCAGTTGATGCAGGCGTG |
| KEAP1_K287R_F | AGGTGCGAGATCCTGCAGT |
| KEAP1_K287R_R | CTGCAGCTGCATCTGCAGGA |
| KEAP1_K303R_F | AGGATCTTCGAGGAGCTCACCCT |
| KEAP1_K303R_R | GACCAGGTAGTCCTTGCAGCGGG |
| KEAP1_K323R_F | AGGGTGGGCCGCCTGATCTACA |
| KEAP1_K323R_R | GGGCGCCCGGCAGGGCATCACCT |
| KEAP1_K615R_F | AGGCAGATTGACCAGCAGAACT |
| KEAP1_K615R_R | CCGGCAGGGCTCCATGGTGACA |
| KEAP1_K551R_F | AGGCACCGGCGAAGTGCCCTGG |
| KEAP1_K551R_R | CATGGGGGCTACGAAAGTCCAC |
| KEAP1_K312R_F | AGGCCCACGCAGGTGATGCCCT |
| KEAP1_K312R_R | GTGCAGGGTGAGCTCCTCGAAG |
| KEAP1_K108R_F | AGGGCCATGTTCACCAACGGGC |
| KEAP1_K108R_R | GAAGACAGGGCTGGATGAGGCC |
| KEAP1_K84R_F | AGGTACCAGGATGCACCGGCCG |
| KEAP1_K84R_R | GACCTGCAGTGTGACGTCACAC |
| KEAP1_K61R_F | AGGCAGGCCTTTGGCATCATG |
| KEAP1_K61R_R | GGTATGATCCTCCAGGGTGTA |
| KEAP1_K131R_F | AGGGTCATGGAGCGCCTCATT |
| KEAP1_K131R_R | GGGGTGGATACCCTCAATGGA |
| KEAP1_K97R_F | AGGGTGGTGCTGGCCTCATCCA |
| KEAP1_K97R_R | GTGGGCCATGAACTGGGCGGC |
| KEAP1_K298/303R_F | TACCTGGTCAGGATCTTCGAGGA |
| KEAP1_K298/303R_R | GTCCCTGCAGCGGGAGTCGGACT |
| KEAP1_K150R_F | AGGTGTGTCCTCCACGTCATGA |
| KEAP1_K150R_R | CTCGCCCATGGAGATGGAGGCC |
