# Supplementary material for: SUMOylation of the ubiquitin ligase component KEAP1 at K39 upregulates NRF2 and its target function in lung cancer cell proliferation
Source: J Biol Chem. 2023 Sep 1;299(10):105215. doi: 10.1016/j.jbc.2023.105215 (PMC10556770; doi:10.1016/j.jbc.2023.105215)
Supplement: Table S2 [file mmc3.docx]

Table S2 primers used for RT-PCR

| Primer | Sequence（5’-3’） |
| --- | --- |
| Human *18S rRNA*_F | TGACTCAACACGGGAAACC |
| Human *18S rRNA*_R | TCGCTCCACCAACTAAGAAC |
| Human *NRF2*_F | CCACTGGTTTCTGACTGGATGT |
| Human *NRF2*_R | TCAGCGACGGAAAGAGTATGA |
| Human *HMOX1*_F | AAGACTGCGTTCCTGCTCAAC |
| Human *HMOX1*_R | AAAGCCCTACAGCAACTGTCG |
| Human *NQO1*_F | GAAGAGCACTGATCGTACTGGC |
| Human *NQO1*_R | GGATACTGAAAGTTCGCAGGG |
| Human *GCLC*_F | GGCACAAGGACGTTCTCAAGT |
| Human *GCLC*_R | CAGACAGGACCAACCGGAC |
| Human *TXNRD1*_F | TAGGACAAGCCCTGCAAGACT |
| Human *TXNRD1*_R | CCCCAATTCAAAGAGCCAATGT |
| Human *AKR1B10*_F | TCAGAATGAACATGAAGTGGGG |
| Human *AKR1B10*_R | TGGGCCACAACTTGCTGAC |
