# Supplementary material for: SUMOylation of the ubiquitin ligase component KEAP1 at K39 upregulates NRF2 and its target function in lung cancer cell proliferation
Source: J Biol Chem. 2023 Sep 1;299(10):105215. doi: 10.1016/j.jbc.2023.105215 (PMC10556770; doi:10.1016/j.jbc.2023.105215)
Supplement: Table S3 [file mmc4.docx]

TableS 3 primers used for chip-PCR

| Primer | Sequence（5’-3’） |
| --- | --- |
| HMOX1-chipF-1 | CCCTGCTGAGTAATCCTTTCCCGA |
| HMOX1-chipR-1 | ATGTCCCGACTCCAGACTCCA |
| NQO1-chipF-1 | CAGAGGCCTCAAAAATCTGG |
| NQO1-chipR-1 | TGGACTCTCTTGGGACGACT |
| GCLC-chipF-1 | GGGCAAAGTCTCAGTCCATC |
| GCLC-chipR-1 | GGCGTGACCACAGAGAAAAT |
| TXNRD1-chipF-1 | CTTTGCTGCCAACTCAATCA |
| TXNRD1-chipR-1 | TCCCACCCCTAGACATTCTG |
|  |  |
